# Macrophage-biomimetic nanomedicine for targeted therapy of abdominal aortic aneurysm via Nrf2/NF-κB pathway

**DOI:** 10.7150/thno.123428

**Published:** 2026-01-01

**Authors:** Qiujie Luo, Wei Meng, Jiahui Wang, Xiang Zhang, Qingbo Meng, Mengjie Hu, Shunbo Wei, Xiaobo Yu, Dawei Deng, Yuqin Zhang, Zixuan Ma, Shentao Li, Shuang Wang, Binhao Zhang, Jingli Ding, Jinping Liu, Jianliang Zhou

**Affiliations:** 1 Department of Cardiovascular Surgery, Zhongnan Hospital of Wuhan University, Wuhan 430071, China.; 2 Hubei Provincial Engineering Research Center of Minimally Invasive Cardiovascular Surgery, Wuhan 430071, China.; 3 Wuhan Clinical Research Center for Minimally Invasive Treatment of Structural Heart Disease, Wuhan 430071, China.; 4 Department of Gastroenterology, Zhongnan Hospital of Wuhan University, Wuhan 430071, China.

**Keywords:** abdominal aortic aneurysm, nanodrug delivery system, metal-organic framework, macrophage polarization, reactive oxygen species

## Abstract

**Rationale:** Abdominal aortic aneurysm (AAA) is a significant cause of death worldwide, with both mortality and incidence rates gradually increasing. Its complex pathological mechanisms hinder drug development. This work aims to develop a biomimetic multifunctional nanoparticle for targeted AAA therapy.

**Methods:** Leveraging the multi-pore property of mesoporous Prussian blue nanoparticles (MPB NPs), we encapsulated the rosiglitazone (RLZ) into MPB NPs to synthesize MPB-RLZ NPs. Then, macrophage-biomimetic nanoparticles (MPB-RLZ@MM NPs) were prepared by coating MPB-RLZ NPs with macrophage membranes (MM). The characterization and reactive oxygen species (ROS)-scavenging ability of MPB-RLZ@MM NPs were evaluated. Next, the biocompatibility and biological functions of MPB-RLZ@MM NPs were evaluated. Finally, we assessed the targeting efficacy and therapeutic efficacy of MPB-RLZ@MM NPs *in vivo*.

**Results:** MPB-RLZ@MM NPs reduced ROS levels in human umbilical vein endothelial cells (HUVECs) and apoptosis in vascular smooth muscle cells (VSMCs). MPB-RLZ@MM NPs inhibited M1-like macrophage polarization via the Nrf2/NF-κB pathway. In addition, MPB-RLZ@MM NPs accumulated in dilated aneurysms. After 4 weeks of treatment, MPB-RLZ@MM NPs effectively delayed aneurysm dilation.

**Conclusions**: MPB-RLZ@MM NPs exhibited excellent biosafety and therapeutic efficacy against AAA. This macrophage-biomimetic strategy presents a promising therapeutic approach for AAA treatment.

## Introduction

Abdominal aortic aneurysm (AAA) is a major cause of cardiovascular-related death worldwide, causing 150,000 to 200,000 deaths annually 1-3. For large (> 5.5 cm) AAAs, surgical operation is the sole treatment option 4. However, only regular imaging monitoring is recommended for small AAAs (< 5.5 cm) 5, 6. There is no drug to delay the AAA growth. Statins have been shown to delay aneurysm dilatation in AAA animal models, but clinical evidence is lacking 7. Therefore, it is urgent to explore treatments that effectively delay AAA dilatation.

AAA involves multiple pathological processes, including macrophage infiltration, oxidative stress, and matrix metalloproteinases (MMPs) activation 8. Inflamed endothelial cells release chemokines and overexpress adhesion factors 9-11. These factors recruit immune cells to the middle and outer layers of aortic aneurysms 9-11. Immune cells produce excessive reactive oxygen species (ROS), such as hydrogen peroxide (H_2_O_2_), hydroxyl radical (•OH), and superoxide anion (O_2_^•-^) 12-14. Pathological ROS promotes inflammation and disrupts the functions of endothelial cells and vascular smooth muscle cells (VSMCs). ROS and inflammation form a self-amplifying vicious cycle, and then chronic inflammation is formed under the synergy of macrophages and T lymphocytes. Contractile VSMCs are crucial for vascular elasticity. Under the stimulations of inflammation and ROS, contractile VSMCs transform into synthetic VSMCs and undergo extensive apoptosis. In addition, activated MMPs (particularly MMP-2 and MMP-9) destroy the elastin and collagen in the extracellular matrix, further leading to irreversible dilation 8.

Over the past few decades, nanomedicines have demonstrated significant advantages in the treatment of cardiovascular diseases 15-19. Mesoporous Prussian Blue nanoparticles (MPB NPs), a subclass of metal-organic frameworks (MOFs), can mimic multiple natural antioxidant enzymes such as superoxide dismutase (SOD) and catalase (CAT) 20-22. The enzyme-like activities convert O_2_^•-^ into H_2_O_2_, and then decompose H_2_O_2_ into harmless water (H_2_O) and oxygen (O_2_) 23-25. Additionally, MPB NPs may modulate the immune microenvironment by activating the Nrf2 antioxidant pathway 26, 27. Prussian blue (PB) is an FDA-approved antidote for radioactive cesium poisoning, with excellent biocompatibility 28. Notably, MPB NPs also show great potential for drug delivery due to their high specific surface area and porosity 24. Rosiglitazone (RLZ), a peroxisome proliferator-activated receptor gamma (PPAR-γ) agonist, demonstrates efficacy in inhibiting the NF-κB pathway and diminishing the M1-like macrophage infiltration 29-31. RLZ has been shown to inhibit the dilation and rupture of AAA in mouse models, primarily demonstrated by reduced levels of E-selectin, tumor necrosis factor-α** (**TNF-α), and interleukin-6 (IL-6), and preserved collagen I/II within the vascular wall 32. Leveraging RLZ's anti-inflammatory properties, encapsulating RLZ within MPB NPs seems to be a promising strategy.

Typically, nanomedicines are passively targeted and accumulate in the inflammatory site through enhanced permeability 15, 16. In recent years, the “bionic camouflage” strategy has enabled active targeting to inflamed endothelial cells 11. In particular, macrophage membrane-coated nanoparticles (MM-NPs) have gained increasing interest in cardiovascular diseases 17, 33. Compared with chemical modifications, MM-NPs exhibit greater immune evasion due to the “self-marking” protein CD47 33. Leveraging the innate homing properties of macrophages, the adhesion factors on MM-NPs (such as integrin α4β1) interact with the surface of inflamed endothelial cells via ligand-receptor binding (such as vascular cell adhesion molecule 1, VCAM-1) 15, 16. Cytokine receptors and toxin-binding receptors on the surface of MM-NPs also neutralize excessive pro-inflammatory cytokines and endotoxins 15.

To address the complex pathology of AAAs, we engineered a multifunctional advanced nanomedicine that encapsulated the anti-inflammatory drug RLZ into MPB NPs (MPB-RLZ NPs) and then coated them with MM vesicles to produce MPB-RLZ@MM NPs. The MM protein CD47 on MPB-RLZ@MM NPs promoted evasion of phagocytosis and prolonged systemic circulation. Additionally, the interaction between integrin α4β1 and VCAM-1 may facilitate the homing of MPB-RLZ@MM NPs to the inflamed endothelium in AAAs. Based on its enzyme-like activities, MPB-RLZ@MM NPs converted pathological ROS (H_2_O_2_, •OH, and O_2_^•-^) into H_2_O and O_2_. Also, the continued release of RLZ modulated the balance of the immune microenvironment, ultimately delaying the progression of AAAs through multiple approaches.

## Materials and Methods

### Materials

Potassium ferricyanide (K_3_[Fe(CN)_6_]) was obtained from Aladdin (Shanghai). Poly(vinylpyrrolidone) (PVP, K30) was purchased from Sinopharm Chemical Reagent Co., Ltd (Shanghai). Rosiglitazone (RLZ) was supplied by Macklin (Shanghai). Hydrogen peroxide (H_2_O_2_), bicinchoninic acid assay (BCA) protein assay kit, 1,1'-dioctadecyl-3,3,3',3'-tetramethylindodicarbocyanine,4-chlorobenzenesulfonate salt (DiD) were purchased from Beyotime Biotechnology Co., Ltd (Shanghai). Phosphate-buffered saline (PBS), lipopolysaccharides (LPS), and gel sample loading buffer were obtained from BioSharp Life Sciences Co., Ltd (Beijing). Interferon-γ (IFN-γ) was purchased from MedChemExpress (U.S.A.). CD47 monoclonal antibody was supplied by Proteintech Group, Inc. (Wuhan). Integrin α4 monoclonal antibody and integrin β1 monoclonal antibody were purchased from Cell Signaling Technology (U.S.A.).

### Preparation of rosiglitazone-loaded MPB nanoparticles (MPB-RLZ NPs)

According to the modified hydrothermal method 28, 131.7 mg of K_3_[Fe(CN)_6_], 3 g of PVP, and 40 mL of HCl (0.01 M) were mixed and stirred for 30 min. The mixture was heated (80 °C, 20 h), centrifuged (12,000 rpm, 10 min), washed with ethanol and ultrapure water, and the MPB NPs were harvested. 10 mL of MPB solution (1 mg/mL in water) was slowly added to 1 mL of RLZ solution (10 mg/mL in ethanol). The mixture was then magnetically stirred for 12 h, centrifuged, and washed with ultrapure water to harvest MPB-RLZ NPs.

### Preparation of macrophage membrane-coated nanoparticles (MPB-RLZ@MM NPs)

Macrophage membranes were isolated as previously described 34. Briefly, RAW264.7 macrophages were suspended in pre-cooled buffer for 15 min, then homogenized 30 times, and centrifuged (700 g, 10 min, 4 °C). Subsequently, the supernatant was harvested and centrifuged (13,000 g, 30 min, 4 °C) to obtain MM. Next, the MM was sonicated for 5 min and extruded 30 times via 0.4 μm polycarbonate (PC) membranes (610007-1Ea, Avestin) by a mini-extruder (610000, Avestin). MPB-RLZ NPs and MM vesicles were mixed in PBS buffer, then sonicated for 5 min, and extruded 30 times via 0.2 μm PC membranes to harvest MPB-RLZ@MM NPs.

### Characterization of the nanoparticles

The morphology and geometric particle size of the nanoparticles were observed by scanning electron microscopy (SEM, SU8100, Hitachi) and transmission electron microscopy (TEM, JEM-2100F, Jeol). Average size measurement using the Nano Measurer 1.2 software (Shanghai). The elemental content of MPB-RLZ@MM NPs was analyzed by energy dispersive spectroscopy (EDS, Aztec X-Max 80T, Cryosystems). The crystal structures were investigated by X-ray diffraction (XRD, SmartLab, Rigaku) and Raman spectrum (DXR3xi, Thermo Fisher). The chemical properties were analyzed by Fourier transform infrared spectroscopy (FTIR, Nicolet iS50, Thermo Fisher) and X-ray photoelectron spectroscopy (XPS, ESCALAB 250Xi, Thermo Fisher). The hydrodynamic particle sizes, zeta potential, and polydispersity index (PDI) were measured by Zetasizer Nano ZS90 (Malvern Panalytical). Ultraviolet-visible spectroscopy (UV-vis, UV2600i, Shimadzu) was used to measure the absorption spectrum. The specific surface area was determined using Brunauer-Emmett-Teller (BET, 3Flex, Micromeritics).

### Drug loading capacity

Freeze-dried MPB-RLZ NPs were dissolved in DMSO, stirred for 12 h, and centrifuged (12,000 rpm, 30 min). The supernatant was collected, and the absorbance was measured at 318 nm by UV-vis. Drug loading capacity (DLC%) was measured using a pre-established standard curve for RLZ (dissolved in DMSO). DLC% = mass (drugs) / mass (nanoparticles) × 100%.

### Identification of macrophage membrane proteins

Briefly, MPB-RLZ NPs, MM vesicles, and MPB-RLZ@MM NPs were each mixed with loading buffer and heated (95 °C, 15 min). Next, the samples were loaded onto the Sodium dodecyl sulfate polyacrylamide gel electrophoresis (SDS-PAGE) gel, which was run at 80 V for 30 min, followed by 120 V for 1 h. Finally, the proteins were visualized with Coomassie Brilliant Blue staining (P0017, Beyotime). The proteins CD47 and integrin α4β1 of MPB-RLZ@MM NPs were identified using Western blotting analysis. The sample proteins were separated by SDS-PAGE, then transferred to PVDF membranes (IPVH00010, Millipore), and incubated overnight at 4 °C with primary antibodies, including anti-CD47 (66304-1-Ig, Proteintech), anti-integrin α4 (8440S, CST), and anti-integrin β1 (34971, CST). Next, the membranes were incubated with the secondary antibody for 1 h. Finally, the protein signals were detected using a chemiluminescent imaging system (5200 Multi, Tanon).

### Cumulative drug release

2 mg of MPB-RLZ@MM NPs was dispersed in 1 mL of PBS buffer (2 mg/mL), then loaded into a dialysis bag (MWCO: 3500 Da, Yuanye). The bag was immersed in 40 mL of PBS buffer or H_2_O_2_ buffer (100 μM) and shaken. At varying time intervals, 1 mL of the buffer was collected, and 1 mL of fresh buffer was added. The absorbance was determined at 318 nm, and the cumulative drug release was measured using a pre-established standard curve for RLZ (in PBS buffer).

### ABST^+•^ scavenging activity

Briefly, 1 mL of MPB-RLZ@MM NPs at various concentrations was mixed with 1 mL of ABST^+•^ working solution (S0119, Beyotime) and incubated for 10 min in the dark. The absorbance at 734 nm was measured.

### •OH scavenging activity

The TiO_2_/UV system generated •OH. MPB-RLZ@MM NPs (1 mg/mL) were reacted with •OH for 10 min. The residual •OH levels were captured by DMPO and detected by electron paramagnetic resonance (EPR) spectra. •OH scavenging capacity was evaluated using salicylic acid (SA) as a probe. FeSO_4_ (2 mM), H_2_O_2_ (5 mM), and SA (1.5 mM) were mixed and incubated for 30 min at 37 °C to produce 2-hydroxysalicylic acid. Different concentrations of MPB-RLZ@MM NPs were incubated with 2-hydroxysalicylic acid for 30 min. The absorbance at 510 nm was measured.

### O_2_^•-^ clearance activity

The xanthine/xanthine oxidase (X/XO) system produced O_2_^•-^. MPB-RLZ@MM NPs (1 mg/mL) were reacted with O_2_^•-^ for 10 min. The residual O2^•-^ was captured by DMPO and detected through EPR.

### H_2_O_2_ scavenging activity

MPB-RLZ@MM NPs were incubated with H_2_O_2_ (1 mM). Dissolved oxygen levels were measured every 30 s using a dissolved oxygen meter (JPB-607A, Leici).

### Enzyme-mimicking activities

According to the manufacturer's manual, SOD-like activity (S0101S, Beyotime), CAT-like activity (S0051, Beyotime), and glutathione peroxidase (GPx)-like activity (S0058, Beyotime) were detected.

### Preparation of DiD- and Rhodamine-labeled nanoparticles

We slowly added 5 mL of MPB solution (1 mg/mL in water) to 500 μL of DiD solution (2 mg/mL in DMSO) or Rhodamine (Rho) solution (2 mg/mL in water), and stirred in the dark for 12 h. We centrifuged the mixture and washed three times to obtain bare MPB-DiD or MPB-Rho NPs. We mixed MPB-DiD or MPB-Rho NPs with MM vesicles in PBS buffer, sonicated for 5 min, and co-extruded via 0.2 μm PC membranes to harvest MPB-DiD@MM or MPB-Rho@MM NPs.

### *In vitro* cytotoxicity assessment

RAW264.7 macrophages, HUVECs, and VSMCs (5 × 10^4^ cells) were treated with MPB-RLZ@MM NPs for 24 h and incubated with MTT working solution (C0009S, Beyotime) for 4 h. Then, formazan solvent was added. Finally, the absorbance at 570 nm was detected to assess cytotoxicity.

### *In vitro* blood compatibility assessment

Fresh blood samples were collected from C57BL/6J mice and then centrifuged to acquire red blood cells (RBCs). MPB-RLZ@MM NPs were incubated with RBCs for 4 h, then centrifuged. The hemolysis phenomenon was observed and photographed, and the absorbance at 540 nm was measured.

### *In vitro* macrophage uptake assay

RAW264.7 macrophages (5 × 10^5^ cells) were treated with MPB-Rho or MPB-Rho@MM NPs (100 μg/mL) for 1, 2, 3, or 4 h, and analyzed by CLSM (STELLARIS 5, Leica) and flow cytometry (CytoFlex S, Beckman). Subsequently, LysoTracker staining (C1047S, Beyotime) was used to label lysosomes. RAW264.7 macrophages were incubated with MPB-Rho or MPB-Rho@MM NPs (100 μg/mL) for 4 h, followed by visualization using CLSM.

### *In vitro* endothelial cell binding assay

HUVECs (1 × 10^5^ cells) were pretreated with or without 50 ng/mL TNF-α (Gibco, Thermo Fisher) for 24 h, and then incubated with MPB-Rho or MPB-Rho@MM NPs (100 μg/mL) for 4 h. Cell binding efficiency was assessed using flow cytometry and CLSM. To validate the role of integrin α4β1-VCAM-1 interaction, VCAM-1 antibodies (300 μg/mL) pre-blocked activated HUVECs for 1 h, followed by co-incubation with MPB-Rho@MM NPs for 4 h 34. Finally, HUVECs were visualized by CLSM.

### *In vitro* ROS scavenging assay

MPB NPs exhibit potent ROS scavenging capacity at 100 μg/mL 20. HUVECs or VSMCs (1 × 10^5^ cells) were pretreated with RLZ (10 μM), MPB NPs, or MPB-RLZ@MM NPs (100 μg/mL) for 12 h. Then, the cells were stimulated with H_2_O_2_ (200 μM) for 4 h, and incubated with 2',7'-Dichlorodihydrofluorescein diacetate (DCFH-DA, 10 μM, S0034S, Beyotime) for 30 min. For RAW264.7 macrophages, the cells (5 × 10^5^ cells) were pretreated as described above, then stimulated with LPS (100 ng/mL) and IFN-γ (20 ng/mL) for 24 h, and incubated with DCFH-DA. Finally, the ROS levels were analyzed by flow cytometry and CLSM.

### *In vitro* VSMC apoptosis assessment

VSMCs (5 × 10^5^ cells) were pretreated with RLZ (10 μM), MPB, or MPB-RLZ@MM NPs (100 μg/mL) for 12 h and then treated with H_2_O_2_ (400 μM) for 6 h. The cells were digested and stained with Annexin V-FITC and propidium iodide (PI, C1062S, Beyotime). Finally, samples were immediately analyzed by flow cytometry.

### *In vitro* macrophage polarization and pro-inflammatory cytokines

RLZ (10 μM) has been validated to exert anti-inflammatory effects by shifting M1-like to M2-like macrophage phenotype 35. RAW264.7 macrophages (1 × 10^6^ cells) were treated as described above. Then, the macrophages were fixed and incubated with primary antibodies: anti-iNOS (GB11119, Servicebio) and anti-CD206 (GB125273, Servicebio). Next, the cells were incubated with secondary antibody and visualized by CLSM. For flow cytometry, the macrophages were stained with PE/Cy7-conjugated anti-CD86 antibody (BioLegend) or PE/Cy5.5-conjugated anti-CD206 antibody (BioLegend) in the dark for 30 min. The cells were then analyzed by flow cytometry (CytoFLEX S, Beckman). For pro-inflammatory cytokines analysis, the supernatants of RAW264.7 macrophages were collected. The levels of IL-6 (RK00008, ABclonal), MCP-1 (RK00381, ABclonal), and TNF-α (RK00027, ABclonal) were measured by enzyme-linked immunosorbent assay (ELISA). Finally, total RNA was extracted using an RNA rapid extraction kit (RN07, Aidlab). cDNA synthesis was used with HiScript III RT SuperMix (R323, Vazyme). Gene expression was analyzed on a CFX Connect 96 system (Bio-Rad) using SYBR Green PCR Master Mix (RK21203, ABclonal). The *Gapdh* gene served as the endogenous control. The relative expression of genes was calculated with the 2^(-ΔΔCt). Primers were listed in Table S1.

### Western blotting

The sample proteins were separated by SDS-PAGE as described above, and then transferred to the PVDF membrane, and incubated overnight at 4 °C with primary antibodies, including anti-Nrf2 (AF0639, Affinity), anti-p-IKKβ (2078, CST), anti-IKKβ (A2087, ABclonal), anti-p-P65 (3033, CST), anti-P65 (10745-1-AP, Proteintech), and anti-GAPDH (60004-1-Ig, Proteintech). Next, the membranes were incubated with the secondary antibody for 1 h. Finally, the protein signals were detected using a chemiluminescent imaging system.

### Animals

Male *ApoE^-/-^* and C57BL/6J mice were obtained from Charles River Laboratories. Mice were maintained under SPF conditions with free access to water and food on a 12-h light/dark cycle. The mice were acclimated for 7 days before the experiments.

### *In vivo* pharmacokinetics and tissue distribution

MPB-DiD or MPB-DiD@MM NPs (10mg/kg) were administered intravenously to C57BL/6J mice. Blood samples were collected at various time points and centrifuged (3000 rpm, 10 min, 4 ℃). The residual DiD signals in plasma were quantified using a microplate reader. Major organs (heart, liver, spleen, lung, and kidney) were collected at different time points. The metabolic profiles of MPB-DiD@MM NPs were evaluated by the *in vivo* imaging system (IVIS, PerkinElmer).

### Angiotensin II (Ang II)-induced AAA model

As previously described in our study 36, 12-week-old male *ApoE^-/-^* mice were implanted subcutaneously with osmotic pumps (2004W, RWD Life Science) delivering Ang II (HY-13948; MedChemExpress) at 1000 ng/kg/min for 28 days. Saline pumps served as negative controls. Abdominal aortic diameters were measured at baseline (Day -1) and the endpoint (Day 28) using the Vevo 2100 small animal ultrasound imaging system (Visual Sonics). AAA was defined as a diameter exceeding 50% of the baseline diameter of the abdominal aorta.

### *In vivo* targeting to AAA

Ang II-induced AAA mice were intravenously administered MPB-DiD or MPB-DiD@MM NPs (10 mg/kg). After 8 h, aortas and major organs were collected and analyzed by IVIS. To investigate the cellular localization of the nanoparticles, the AAA tissue sections were stained with F4/80 (macrophage marker). Finally, the sections were analyzed by fluorescence microscopy.

### Therapeutic efficacy in AAA mice

Mice were divided into five groups: (1) sham group, (2) Ang II group, (3) Ang II + free RLZ group, (4) Ang II + bare MPB group, and (5) Ang II + MPB-RLZ@MM group. The Ang II + free RLZ group was given an oral free RLZ solution. The Ang II + bare MPB group received intravenously MPB NPs. The Ang II + MPB-RLZ@MM group was intravenously administered MPB-RLZ@MM NPs. RLZ (10 mg/kg/day) was administered consistently, as in previous studies 32. The dosage of RLZ has been reported to effectively inhibit the progression of AAA 32. MPB NPs (10 mg/kg) have demonstrated therapeutic effects in inflammatory disease models 24, 37, 38. Bare MPB NPs (10 mg/kg) and MPB-RLZ@MM NPs (10 mg/kg) were administered at 3-day intervals from the second day until the endpoint of the 28-day experimental period.

### Histology evaluation

The aortic tissues were carefully isolated under a dissecting microscope. For histopathological analysis, aortic tissue sections were analyzed by H&E (hematoxylin and eosin), Masson's trichrome, and EVG (Elastic Van Gieson) staining. For immunohistochemistry (IHC) staining, tissue sections were incubated with primary antibodies: anti-F4/80 (GB113373, Servicebio) and anti-α-SMA (GB111364, Servicebio). The sections were then incubated with secondary antibodies and visualized using 3,3'-diaminobenzidine (DAB). Quantitative analysis of IHC staining was performed using ImageJ 1.8.0 (Wayne Rasband, U.S.A.) equipped with the IHC toolbox. Immunofluorescence staining was performed with the following primary antibodies: anti-iNOS (GB13594, Servicebio) and anti-CD206 (GB113497, Servicebio). Dihydroethidium (DHE, S0063, Beyotime) staining was used to quantify ROS levels in the aorta. The extent of elastin degradation was graded according to established criteria 39 as follows: grade 1, no degradation; grade 2, sporadic breaks in elastic layers; grade 3, multiple discontinuities within elastic lamellae; grade 4, severe fragmentation or loss of elastin. The levels of IL-6 (RK00008, Abclonal), TNF-α (RK00027, Abclonal), and MCP-1 (RK00381, Abclonal) were measured using ELISA kits. RLZ concentrations were detected in the aortas and major organs using a Liquid Chromatograph Mass Spectrometer (LC-MS, TripleQTOF5600+, Absciex). Specifically, tissue samples were mixed with extraction solvent (methanol: acetonitrile, v/v = 1:1) and homogenized (60 Hz, 2 min). The homogenized mixture was incubated (-20 °C, 30 min), and then centrifuged (13,000 rpm, 15 min). Collect the supernatant and freeze-dry overnight. The freeze-dried samples were resuspended with a solvent (methanol: water, v/v = 9:1). The samples were incubated (4 °C, 30 min), and then centrifuged (13,000 rpm, 15 min). Finally, the supernatant was detected by LC-MS.

### Therapeutic efficacy in established small AAA

The small AAA model was established by 2-week Ang II-pump perfusion without therapeutic intervention. After 2 weeks, Mice were divided into three groups: (1) sham group, (2) Ang II group, (3) Ang II + MPB-RLZ@MM group. The dosage of MPB-RLZ@MM NPs was as described above. At 28 days, the aortic diameter was measured by ultrasound. The mice were euthanized for histological analysis.

### Bulk RNA sequencing

Total RNA was extracted using Trizol (Thermo Fisher). The RNA samples were then purified and used for library construction. The sequencing data were analyzed with the online analysis platform (https://www.genescloud.cn/home). Principal component analysis (PCA) was conducted to cluster samples with similar expression profiles through linear transformation. Subsequently, bidirectional hierarchical clustering of genes and samples was performed using the Pheatmap and ComplexHeatmap packages in R. Differentially expressed genes (DEGs) were visualized with a volcano plot (fold change > 2 and *P*-adj < 0.05). Finally, Gene Ontology (GO) and Kyoto Encyclopedia of Genes and Genomes (KEGG) were used to analyze gene enrichment. Additionally, Gene Set Enrichment Analysis (GSEA) was used to assess the enrichment of predefined gene sets within the expression profiles.

### *In vivo* RLZ concentration in non-specific organs

Male C57BL/6J mice were administered with RLZ (2mg/kg) orally or MPB-RLZ@MM NPs (2 mg/kg RLZ) intravenously. After 6 h, the major organs were collected. LC-MS measured RLZ concentrations of tissue samples.

### *In vivo* biocompatibility assessment

Male C57BL/6J mice were administered intravenously with MPB-RLZ@MM NPs (10 mg/kg), while negative controls received an equivalent volume of saline. Blood samples and major organs were harvested on Days 3, 7, 14, 28, and 56. The automatic blood cell analyzer (BC-2800vet, Mindray) was used to measure blood cell counts. The biochemistry analyzer (Chemray-240, Rayto) was used to detect biochemical markers. The main organs were fixed and then analyzed by H&E staining.

### Statistical analysis

Data were presented as means ± standard deviation (SD). One-way analysis of variance (ANOVA) and Student's *t*-test were used for comparisons in the GraphPad Prism 9.5.0 (GraphPad Software, California, U.S.A.). Differences were considered statistically significant when *P* < 0.05.

## Results

### Characterization of MPB-RLZ NPs

**Figure 1:**
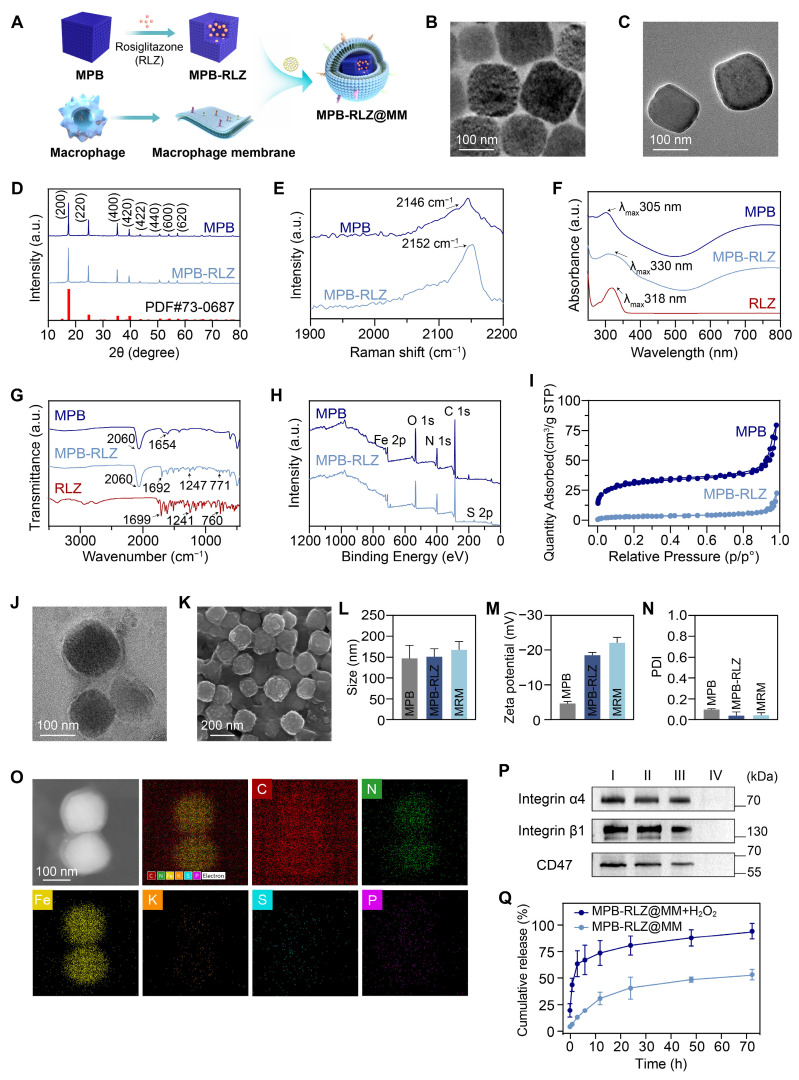
** Characterization of MPB-RLZ@MM NPs.** (**A**) Schematic diagram depicting the synthesis of MPB-RLZ@MM NPs. (**B-C**) TEM images of MPB and MPB-RLZ NPs. Scale bar = 100 nm. (**D**) XRD pattern of MPB and MPB-RLZ NPs. (**E**) Raman spectra of MPB and MPB-RLZ NPs. (**F**) UV-vis spectra of MPB NPs, RLZ, and MPB-RLZ NPs. (**G**) FTIR spectra of MPB NPs, RLZ, and MPB-RLZ NPs. (**H**) XPS spectra of MPB and MPB-RLZ NPs. (**I**) N_2_ adsorption-desorption isotherms of MPB and MPB-RLZ NPs. (**J**) TEM image of MPB-RLZ@MM NPs. Scale bar = 100 nm. (**K**) SEM image of MPB-RLZ@MM NPs. Scale bar = 200 nm. (**L**) Average geometric particle size of the nanoparticles. MPB NPs (n = 44) vs MPB-RLZ NPs (n = 55) vs MPB-RLZ@MM (MRM) NPs (n = 41). Data were presented as mean ± SD. (**M-N**) Zeta potential and PDI of the nanoparticles (n = 3, mean ± SD). (**O**) Elemental mapping of MPB-RLZ@MM NPs. Scale bar = 100 nm. (**P**) Western blotting analysis of CD47 and integrin α4β1 protein expression. I: macrophages; II: macrophage membrane; III: MPB-RLZ@MM NPs; IV: MPB-RLZ NPs. (**Q**) Cumulative release of free RLZ from MPB-RLZ@MM NPs in PBS or H_2_O_2_ buffer (n = 3, mean ± SD).

MPB NPs were synthesized using a modified hydrothermal method 28, and MPB-RLZ NPs were then prepared through a solution exchange method leveraging RLZ's hydrophobic property. SEM and TEM images (Figure 1B-C; Figure S1) demonstrated that both MPB and MPB-RLZ NPs exhibited cubic morphology, with average particle sizes of about 146.5 nm for MPB NPs and 150.2 nm for MPB-RLZ NPs (Figure 1L). Zeta potential analysis (Figure 1M) showed the surface potentials of MPB-RLZ NPs (-18.4 mV) and MPB NPs (-4.7 mV). XRD analysis (Figure 1D) characterized that the peaks for both MPB and MPB-RLZ NPs appeared at 17.4° (200), 24.8° (220), 35.2° (400), 39.4° (420), 43.5° (422), 50.7° (440), 53.9° (600), 67.2° (620), and 68.9° (640), matching the standard diffraction card JCPDS 73-0687. Raman spectrum (Figure 1E) showed a peak occurring nearly 2150 cm^-1^ (C≡N) in both MPB and MPB-RLZ NPs. UV-vis (Figure 1F) demonstrated that MPB NPs exhibited an absorption peak at 305 nm, and RLZ displayed a peak at 318 nm. After RLZ loading, the absorption peak of MPB-RLZ NPs appeared at 330 nm. FTIR spectrum (Figure 1G) showed peaks of MPB NPs appeared at 2060 cm^-1^ (Fe^2+^-CN-Fe^3+^) and 1654 cm^-1^ (C=O), and peaks of RLZ occurred at 1699 cm^-1^ (C=O) and 1241 cm^-1^ (C-O-C). The FTIR spectra of MPB-RLZ NPs exhibited peaks corresponding to both RLZ and MPB NPs. XPS (Figure 1H) showed that an S 2p peak appeared in the 163-165 eV range for MPB-RLZ NPs, providing direct evidence of RLZ loading. The sulfur content (Table S2) increased from 0% (MPB NPs) to 2.1% (MPB-RLZ NPs). Concurrently, high-resolution Fe 2p spectra (Figure S2-3) showed decreased intensities of the Fe 2p_3/2_ (708.1 eV) and Fe 2p_1/2_ (721.1 eV) peaks in MPB-RLZ NPs. N_2_ adsorption-desorption isotherm analysis (Figure 1I) revealed a significant decrease in the BET specific surface area of MPB-RLZ NPs (11.18 m^2^/g) compared to MPB NPs (113.03 m^2^/g). The drug loading capacity of MPB-RLZ NPs is approximately 210.25 μg/mg (Figure S4). Together, these results confirmed the successful loading of RLZ into MPB NPs.

### Identification of macrophage membrane proteins

MPB-RLZ NPs and MM vesicles were co-extruded to synthesize MPB-RLZ@MM NPs (Figure 1A). SEM and TEM images (Figure 1J-K) showed the encapsulation of MPB-RLZ NPs within the MM, revealing a clear core-shell structure. The average particle sizes of MPB-RLZ@MM NPs (165.7 nm) increased by 15.49 nm compared to MPB-RLZ NPs (Figure 1L), and the surface potential of MPB-RLZ@MM NPs (-20.9 mV) decreased by 3.4 mV (Figure 1M). PDI of MPB-RLZ@MM NPs was below 0.1 (Figure 1N), indicating excellent colloidal stability. Elemental mapping images (Figure 1O) showed the co-localization of Fe and K (Prussian blue core) with P (MM phospholipid biomarker). EDS elemental analysis further quantified the P element content at 0.5 wt% (Figure S5; Table S3). Western blotting and SDS-PAGE (Figure 1P; Figure S6) analysis verified the retention of membrane proteins such as CD47 and integrin α4β1. Furthermore, MPB-RLZ@MM NPs demonstrated consistent stability for 7 days in PBS buffer and Dulbecco's modified Eagle's medium (DMEM) containing 10% fetal bovine serum (FBS) (Figure S7). Next, the drug release kinetics of MPB-RLZ@MM NPs showed that the cumulative release of free RLZ was 51.33% in the PBS buffer and 88.51% in the H_2_O_2_ buffer after 72 h of incubation (Figure 1Q).

### ROS scavenging ability

**Figure 2:**
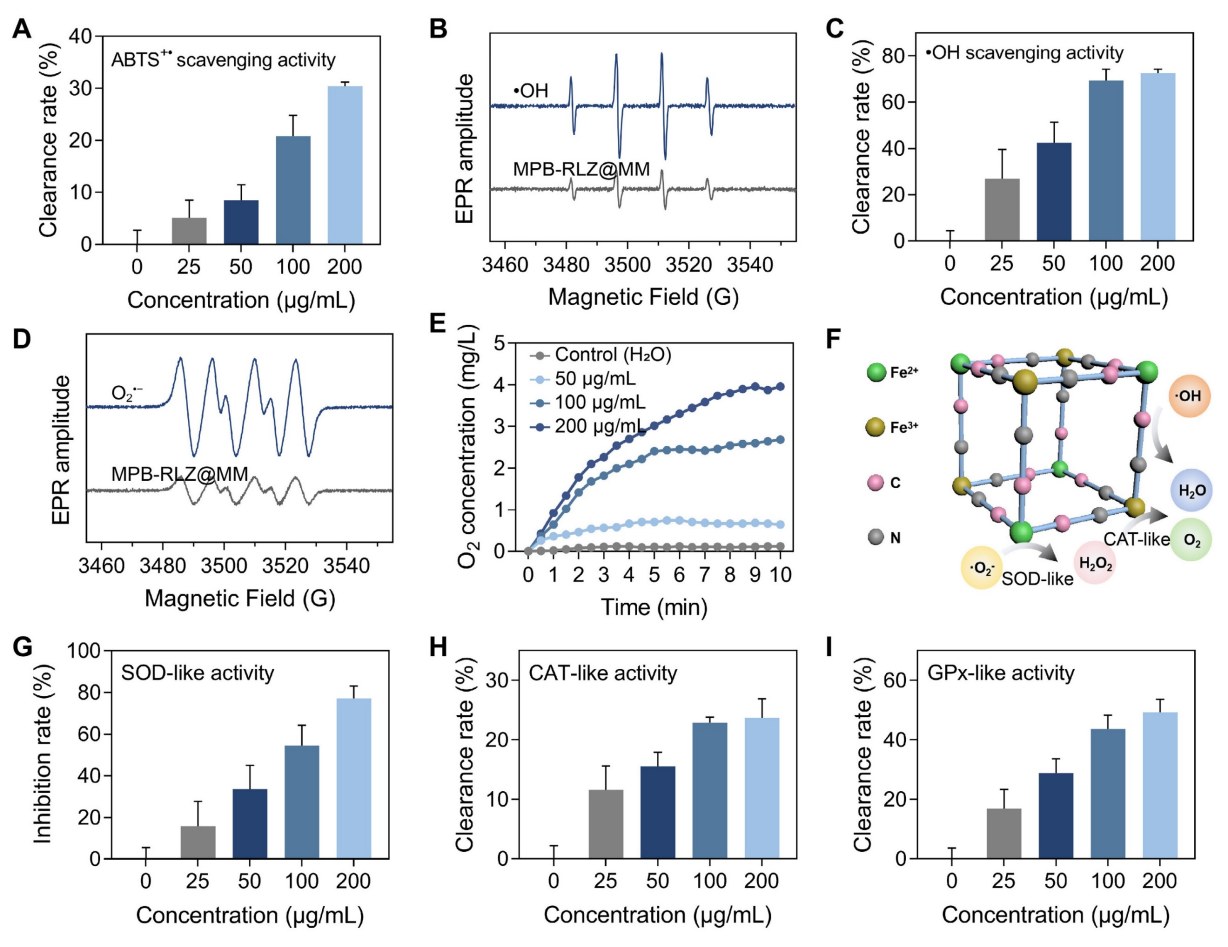
** ROS scavenging activities.** (**A**) The total antioxidant capacity of MPB-RLZ@MM NPs was assessed by the ABTS^+•^ scavenging assay (n = 3, mean ± SD). (**B**) The •OH scavenging effect of MPB-RLZ@MM NPs was evaluated by EPR spectra. (**C**) The •OH scavenging efficiency of MPB-RLZ@MM NPs (n = 3, mean ± SD). (**D**) The O_2_^•-^ scavenging effect of MPB-RLZ@MM NPs was detected via EPR spectra. (**E**) The dissolved oxygen levels of MPB-RLZ@MM NPs were measured through the H_2_O_2_ decomposition assay. (**F**) Schematic diagram illustrating ROS scavenging mechanisms of MPB-RLZ@MM NPs. (**G-I**) The enzyme-mimicking assays included SOD-like, CAT-like, and GPx-like activities (n = 3, mean ± SD).

The radical scavenging capacity was evaluated using the 2,2'-azino-bis (3-ethylbenzothiazoline-6-sulfonic acid) (ABTS) free radicals. MPB-RLZ@MM NPs showed the ABTS**^+•^** scavenging efficiencies of approximately 5.13% (25 μg/mL), 8.48% (50 μg/mL), 20.78% (100 μg/mL), and 30.34% (200 μg/mL) (Figure 2A). EPR spectra revealed significantly reduced •OH levels in the MPB-RLZ@MM group (Figure 2B). Furthermore, •OH scavenging efficiency was determined using the SA/•OH system (Figure 2C), with scavenging rates of 26.90% (25 μg/mL), 42.48% (50 μg/mL), 69.32% (100 μg/mL), and 72.55% (200 μg/mL). O_2_^•-^ scavenging ability was further evaluated using an X/XO system. Similarly, MPB-RLZ@MM treatment markedly reduced O_2_^•-^ levels by EPR (Figure 2D). The dissolved oxygen assay (Figure 2E) showed that the different concentrations of MPB-RLZ@MM NPs increased dissolved O_2_ concentration. The mechanism of free radical scavenging by MPB-RLZ@MM NPs was achieved via SOD-like and CAT-like activities (Figure 2F). Furthermore, the water-soluble tetrazolium dye (WST-8) assay (Figure 2G) showed the SOD-like activity of MPB-RLZ@MM NPs increasing from 15.84% (25 μg/mL) to 77.22% (200 μg/mL). Concurrently, MPB-RLZ@MM NPs also showed strong CAT-like and GPx-like activities (Figure 2H-I).

### *In vitro* immune evasion and cell adhesion

**Figure 3:**
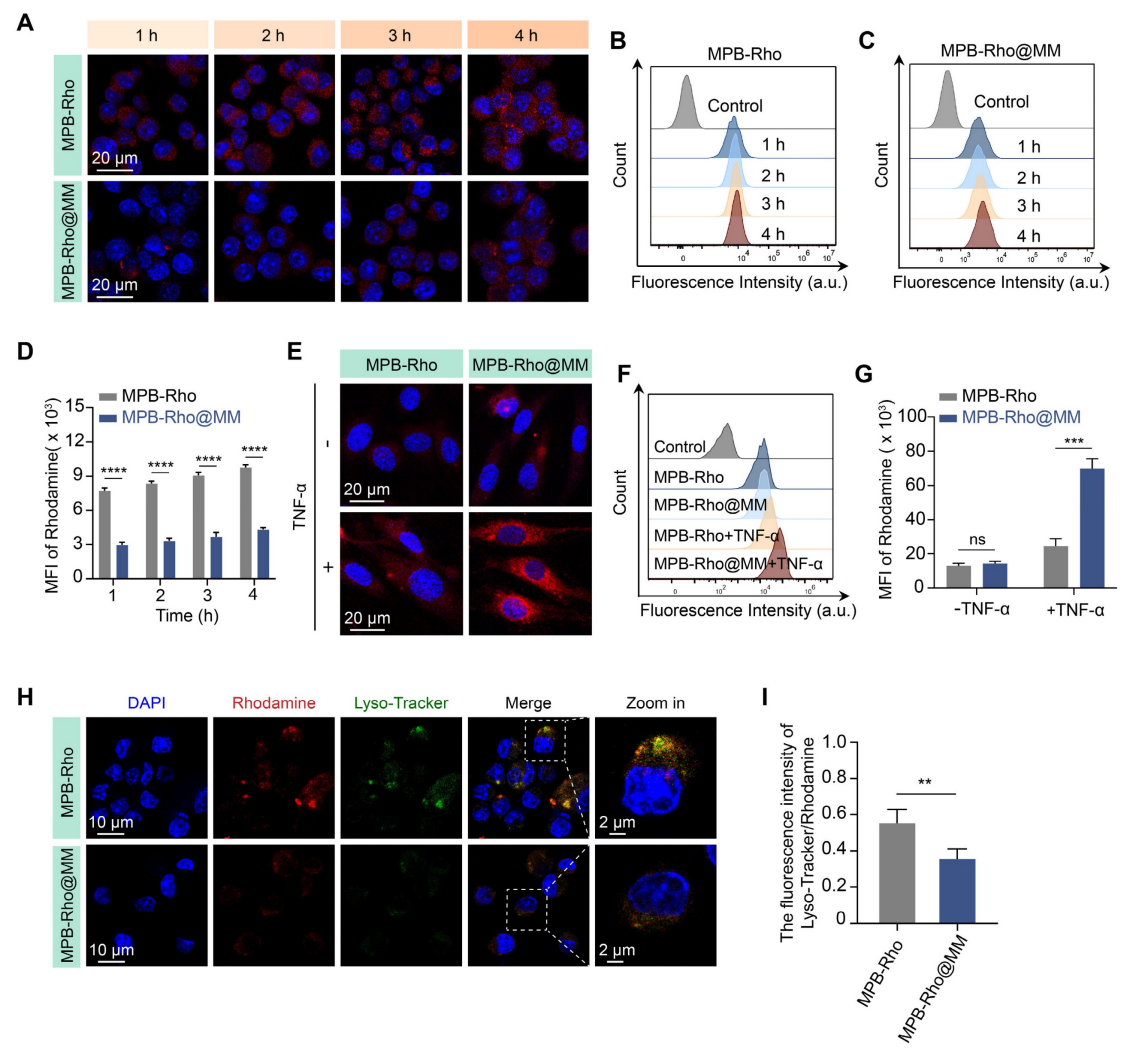
** Macrophage cellular uptake and endothelial cell adhesion.** (**A**) CLSM imaging of RAW264.7 macrophages internalization of MPB-Rho or MPB-Rho@MM NPs at different time points (1, 2, 3, and 4 h). Scale bar = 20 µm. (**B-C**) Flow cytometric analysis of cellular uptake in RAW264.7 macrophages. (**D**) Quantitative analysis of uptake efficiency in RAW264.7 macrophages via flow cytometry. (**E-F**) CLSM images and flow cytometry analysis of MPB-Rho or MPB-Rho@MM NPs internalization in endothelial cells with or without TNF-α activation. Scale bar = 20 µm. (**G**) Quantitative flow cytometry assessment of endothelial cell internalization. (**H-I**) Co-localization of MPB-Rho or MPB-Rho@MM NPs with lysosomes in RAW264.7 macrophages. Scale bar = 10 µm. Data were presented as the mean ± SD from three independent experiments. “*ns*”, no significance, **p < 0.01, ***p < 0.001, ****p < 0.0001, as determined by Student's *t*-test.

To evaluate the immune evasion ability of the MM-NPs, we synthesized Rho-labeled MPB-Rho and MPB-Rho@MM NPs. CLSM results showed red fluorescence (Rho) signals in both MPB-Rho and MPB-Rho@MM groups. The Rho signals in the MPB-Rho@MM group were lower than those in the MPB-Rho group (Figure 3A). Flow cytometry further confirmed the reduction of Rho signals in the MPB-Rho@MM group (Figure 3B-D), indicating that MM-NPs reduced phagocytosis. Then, we evaluated the lysosome escape using LysoTracker as a probe. The MPB-Rho group showed the co-localization of Rho (red) and LysoTracker (green) signals. However, the LysoTracker signals in the MPB-Rho@MM group were weakened (Figure 3H-I), indicating reduced lysosomal transport. To evaluate the targeting of MM-NPs to inflamed endothelial cells, we further co-incubated TNF-α-activated HUVECs with MPB-Rho@MM NPs. CLSM imaging showed increased Rho signals within activated HUVECs of the MPB-Rho@MM group. (Figure 3E). Flow cytometry confirmed that the Rho fluorescence intensity in the MPB-Rho@MM group was 2.8 times higher than that in the MPB-Rho group (Figure 3F-G). To verify the role of integrin α4β1-VCAM-1 interaction, we performed the VCAM-1 antibody blocking assay. CLSM imaging (Figure S8) showed reduced Rho signals in the VCAM-1 blocking group, indicating that the MM-NPs binding was blocked. This result suggests that the integrin α4β1-VCAM-1 interaction may play a crucial role in mediating the binding of nanomedicine.

### *In vitro* cellular ROS scavenging

**Figure 4:**
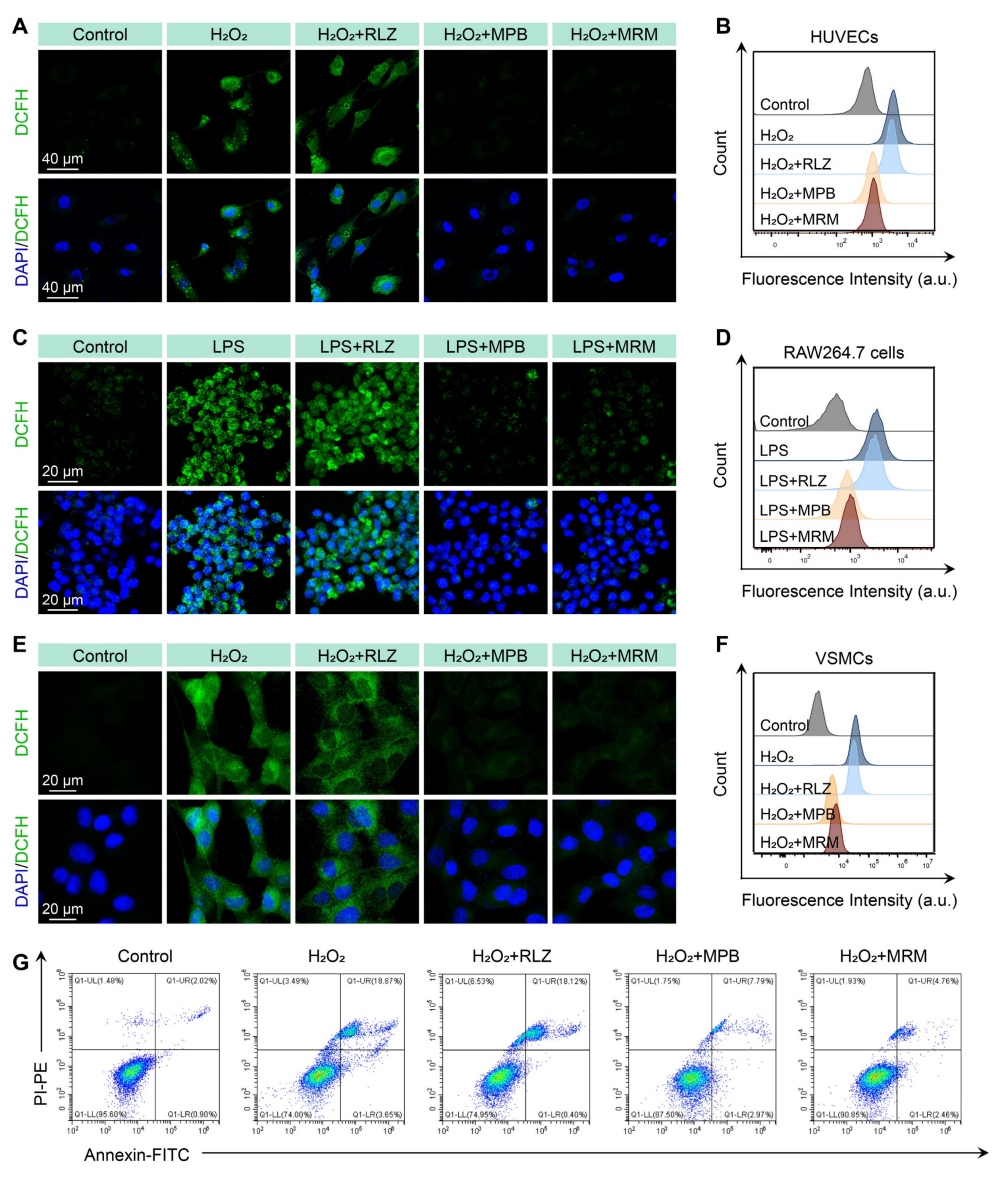
** MPB-RLZ@MM NPs suppress intracellular ROS levels and apoptosis.** (**A-B**) CLSM and flow cytometry analysis of H_2_O_2_-induced ROS levels in HUVECs treated with RLZ, MPB NPs, or MRM (MPB-RLZ@MM) NPs. Scale bar = 40 µm. (**C-D**) CLSM and flow cytometry of LPS/IFN-γ-induced ROS levels in RAW264.7 macrophages with various treatments. Scale bar = 20 µm. (**E-F**) CLSM and flow cytometry of H_2_O_2_-triggered ROS levels in VSMCs with different treatments. Scale bar = 20 µm. (**G**) Flow cytometry analysis of VSMC apoptosis induced by H_2_O_2_ using Annexin V/PI staining.

We established an oxidative stress model in HUVECs using H_2_O_2_ stimulation, with DCFH-DA as a ROS probe. CLSM images (Figure 4A) revealed green fluorescence (DCFH) in the H_2_O_2_ group, indicating intense oxidative stress in HUVECs. The RLZ treatment did not significantly decrease the fluorescence intensity. In contrast, the MPB and MPB-RLZ@MM treatments significantly reduced DCFH signal intensity. Flow cytometry analysis further confirmed significantly lower ROS levels in the MPB and MPB-RLZ@MM treatments (Figure 4B; Figure S9). Excessive ROS promotes M1-like macrophage polarization and VSMC apoptosis. We further assessed ROS levels in LPS/IFN-γ-stimulated RAW264.7 macrophages with different treatments. CLSM images and flow cytometry (Figure 4C-D; Figure S9) showed intense fluorescence intensity in the LPS group, while decreased fluorescence was observed in the MPB-RLZ@MM group. Next, we examined the effect of MPB-RLZ@MM on H_2_O_2_-induced VSMC apoptosis. CLSM imaging and flow cytometry (Figure 4E-F; Figure S9) confirmed markedly lower ROS levels for the MPB-RLZ@MM treatment. Apoptosis analysis (Figure 4G; Figure S9) showed that the MPB-RLZ@MM treatment significantly reduced the apoptosis rate of VSMCs. Collectively, these findings demonstrate that MPB-RLZ@MM NPs are effective against free radical-induced cellular damage.

### *In vitro* macrophage polarization and inflammatory modulation

**Figure 5:**
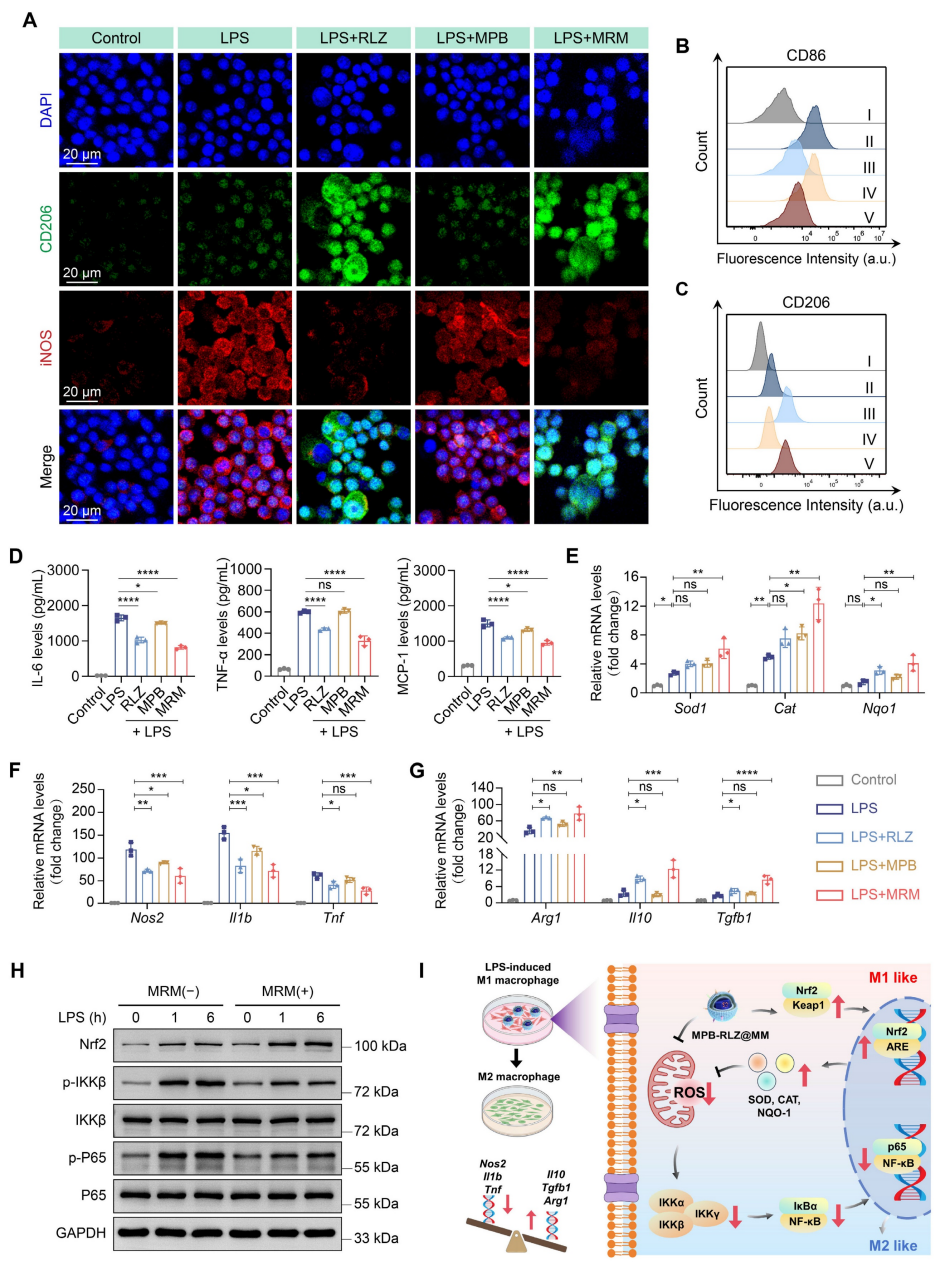
**Effects on macrophage polarization and pro-inflammatory cytokines.** (**A**) CLSM images of LPS/IFN-γ-stimulated macrophage polarization treated with free RLZ, MPB NPs, and MRM (MPB-RLZ@MM) NPs. Scale bar = 20 µm. (**B-C**) Flow cytometry analysis of CD86^+^ and CD206^+^ macrophages under different treatments. I: control; II: LPS; III: LPS + RLZ; IV: LPS + MPB; V: LPS + MRM. (**D**) Levels of IL-6, TNF-α, and MCP-1 in supernatant of RAW264.7 macrophages assessed by ELISA. (**E**) Relative mRNA expression levels of antioxidant genes (*Sod1*, *Cat*, and *Nqo1* genes) measured by qPCR. (**F-G**) Relative mRNA expression of pro-inflammatory genes (*Nos2*, *Il1b*, and *Tnf* genes) and anti-inflammatory genes (*Arg1*, *Il10*, and *Tgfb1* genes). (**H**) Western blotting analysis of Nrf2, p-IKKβ, IKKβ, p-P65, and P65 protein expression. (**I**) The mechanism diagram of anti-inflammatory and anti-oxidative stress effects of MPB-RLZ@MM NPs in macrophages. Data were expressed as the mean ± SD of three independent experiments. “*ns*”, no significance, *p < 0.05, **p < 0.01, ***p < 0.001, and ****p < 0.0001 by ANOVA with Dunnett's multiple comparisons test.

To investigate the effects of MPB-RLZ@MM NPs on macrophage polarization, RAW264.7 macrophages were stimulated with LPS/IFN-γ. CLSM imaging (Figure 5A) revealed strong red fluorescence (iNOS, M1 marker) and weak green fluorescence (CD206, M2 marker) in the LPS group, indicating a significant M1-like macrophage phenotype. The MPB group showed no significant change compared to the LPS group. In contrast, the RLZ and MPB-RLZ@MM groups showed decreased iNOS signals and increased CD206 signals, confirming a shift from M1-like toward M2-like macrophage phenotype. Flow cytometry (Figure 5B-C; Figure S10) further confirmed that free RLZ and MPB-RLZ@MM treatments decreased CD86 (M1 marker) levels while increasing CD206 levels compared to LPS treatment. ELISA results (Figure 5D) showed that the LPS/IFN-γ stimulation increased the levels of IL-6, TNF-α, and MCP-1 in the supernatant of RAW264.7 macrophages. In contrast, Free RLZ and MPB-RLZ@MM treatments suppressed the levels of pro-inflammatory cytokines. MPB-RLZ@MM treatment increased the expression of antioxidant stress genes, such as *Cat*, *Sod1*, and *Nqo1* genes, while decreasing the expression of pro-inflammatory genes, including *Tnf*, *Il1b*, and *Nos2* genes (Figure 5E-F). Additionally, MPB-RLZ@MM treatment boosted the expression of anti-inflammatory genes, including *Tgfb1*, *Il10*, and *Arg1* genes (Figure 5G). These findings indicated that MPB-RLZ@MM NPs suppressed oxidative stress and M1-like phenotype while promoting M2-like phenotype.

Nuclear factor erythroid 2-related factor 2 (Nrf2) plays a key role in regulating oxidative stress. Normally, Nrf2 binds to its inhibitory protein (kelch-like ECH-associated protein 1, Keap1) and is localized in the cytoplasm. Exposure to oxidative stress causes Nrf2 to dissociate from the Nrf2-Keap1 complex and rapidly translocate to the nucleus. Within the nucleus, Nrf2 binds to the antioxidant response element (ARE) and upregulates the expression of multiple antioxidant genes. The reduction in ROS further inhibits the activation of the NF-κB pathway. Therefore, Nrf2 is a promising target for inhibiting oxidative stress and inflammation. Western blotting analysis showed that LPS/IFN-γ stimulation upregulated the Nrf2 protein expression at 1 h and 6 h, suggesting the activation of the Nrf2 antioxidant pathway. MPB-RLZ@MM treatment further increased the Nrf2 expression (Figure 5H; Figure S11). LPS/IFN-γ stimulation also upregulated the expressions of phosphorylated IKKβ (p-IKKβ) and phosphorylated P65 (p-P65). However, MPB-RLZ@MM treatment reduced the expressions of p-IKKβ and p-p65, suggesting inhibition of the NF-κB pathway (Figure 5H; Figure S11). Next, we investigated the relationship between the reduction of ROS and pro-inflammatory cytokines in RAW264.7 macrophages. CLSM and ELISA analysis (Figure S12) revealed that MPB-RLZ@MM treatment reduced ROS levels while also decreasing the levels of pro-inflammatory cytokines (IL-6, TNF-α, MCP-1). In summary, MPB-RLZ@MM NPs may promote Nrf2 activation and nuclear translocation in macrophages, thereby upregulating antioxidant stress genes and anti-inflammatory genes while suppressing pro-inflammatory genes. Subsequently, MPB-RLZ@MM NPs eliminated excessive ROS and inhibited the NF-κB pathway. The schematic diagram of the mechanism is shown in Figure 5I.

### *In vivo* pharmacokinetics and tissue distribution

To investigate the pharmacokinetics of MM-NPs, we synthesized DiD-labeled nanoparticles (MPB-DiD and MPB-DiD@MM NPs). After mice were intravenously injected with DiD-labeled nanoparticles, blood samples were collected at different time points. The MPB-DiD group showed that the DiD signals decreased rapidly and were almost undetectable after 12 h. However, the MPB-DiD@MM group still retained approximately 20% of the initial DiD signal intensity at 12 h (Figure S13). This result suggested that MM-NPs improved the longer blood circulation time. Then, the metabolism profiles of MM-NPs were evaluated by IVIS. Due to the large size effect (> 100 nm), MPB-DiD@MM NPs were mainly distributed in the liver and spleen tissues (Figure S14). IVIS imaging showed that MPB-DiD@MM NPs were cleared in the body within approximately 7 to 10 days (Figure S14).

### *In vivo* targeting efficacy of AAA

**Figure 6:**
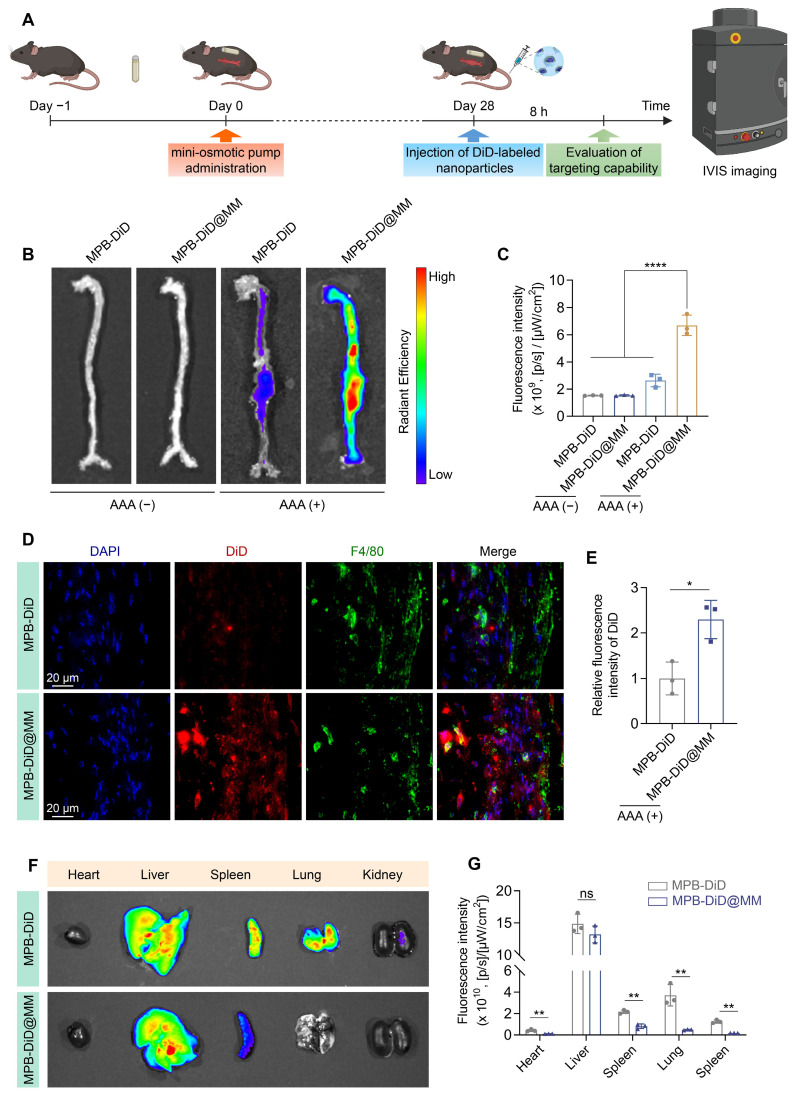
** Targeted delivery to AAA in *ApoE⁻^/^⁻* mice.** (**A**) Schematic diagram illustrating the experimental procedure for targeted delivery of MPB-DiD@MM NPs to AAA. (**B-C**) Representative images of *ex vivo* aortic fluorescence imaging detected by IVIS, along with the quantified fluorescence intensity. (**D-E**) Representative imaging of AAA tissue sections showing DiD and F4/80 fluorescence signals, accompanied by quantitative analysis. Scale bar = 20 µm. (**F-G**) Tissue distribution of DiD-labeled nanoparticles was assessed via IVIS, and the fluorescence intensity was measured in major organs. Data were expressed as the mean ± SD of three independent experiments. “*ns*”, no significance, *p < 0.05, **p < 0.01, and ****p < 0.0001 as determined by ANOVA with Dunnett's multiple comparisons test or Student's *t*-test.

To evaluate the AAA targeting ability of MM-NPs, we established the Ang II-induced AAA model. After 4 weeks of Ang II infusion, the mice were intravenously injected with MPB-DiD or MPB-DiD@MM NPs. The aortic tissues were collected after 8 h and analyzed by IVIS (Figure 6A). The MPB-DiD group showed weak DiD signals in the dilated aneurysm. The MPB-DiD@MM group showed stronger DiD signals compared to the MPB-DiD group (Figure 6B-C), suggesting active targeting. In addition, we evaluated the co-localization of the nanoparticles and macrophages by F4/80 staining. The immunofluorescence results showed that the co-localization of DiD signals (red) with F4/80^+^ macrophages (green) was more significant (Figure 6D-E) in the MPB-DiD@MM group. IVIS imaging revealed that the MPB-DiD@MM group exhibited lower DiD signals in the major organs (Figure 6F-G), suggesting that MM-NPs reduced off-target effects.

### *In vivo* therapeutic efficacy in AAA

**Figure 7:**
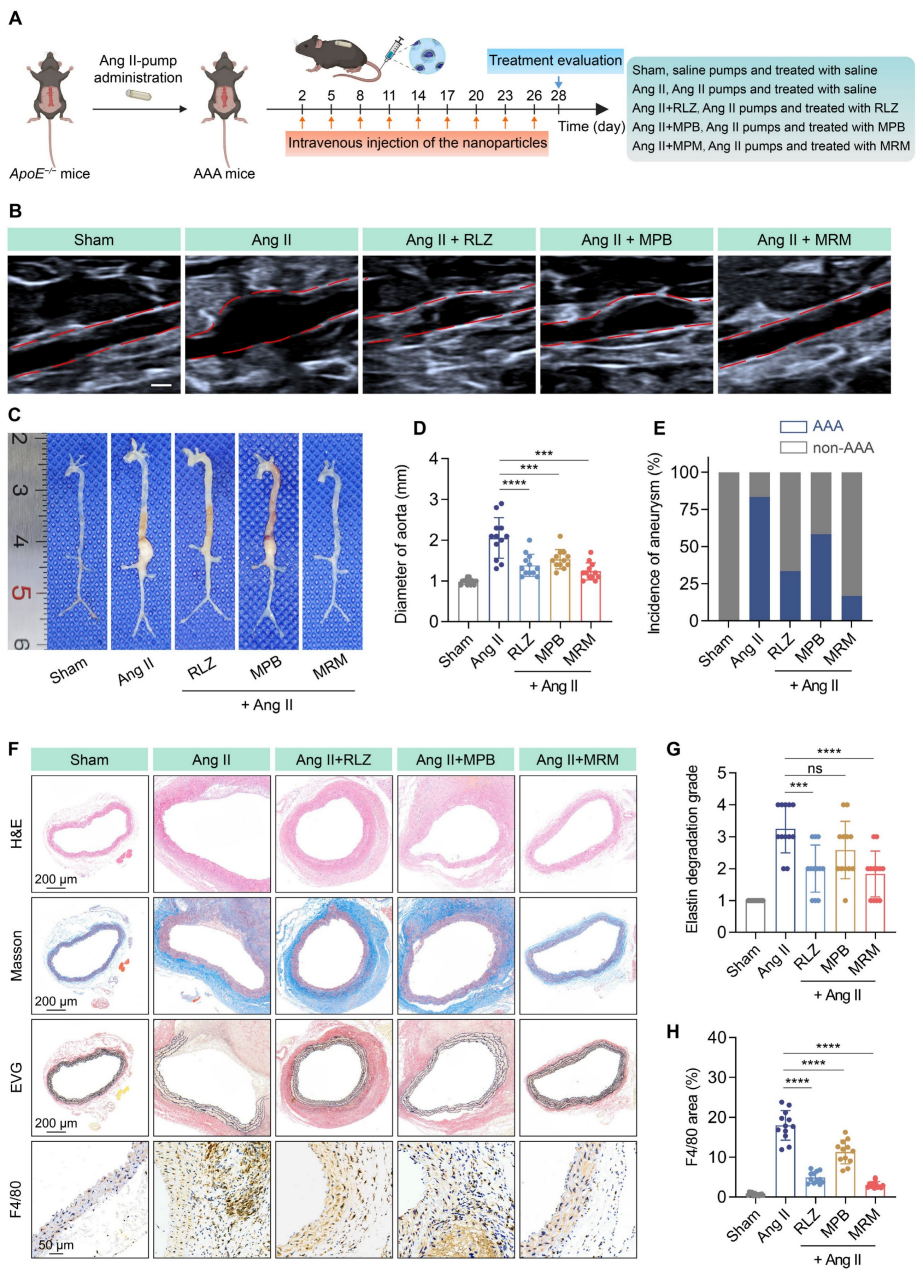
** Therapeutic efficacy in the AAA model.** (**A**) Experimental design schematic of MPB-RLZ@MM NPs in therapeutic experiment. (**B**) Representative ultrasound images of the abdominal aorta with red dashed lines outlining the aortic contours. MRM means MPB-RLZ@MM NPs. Scale bar = 1 mm. (**C**) Representative images of the aorta from the aortic arch to the iliac arteries in different treatment groups. (**D**) Maximum aortic diameter at different treatment groups (n = 12, mean ± SD). (**E**) Incidence rate of aneurysm formation (defined as more than a 50% increase from the baseline diameter, n = 12). (**F**) Histopathological analysis of H&E (scale bar = 200 µm), Masson's trichrome (scale bar = 200 µm), EVG (scale bar = 200 µm), and F4/80 staining (scale bar = 50 µm). (**G-H**) Elastin degradation score and F4/80^+^ macrophage infiltration area in aortic sections (n = 12, mean ± SD). *ns*, no significance, ***p < 0.001, and ****p < 0.0001 by ANOVA with Dunnett's multiple comparisons test.

Subsequently, we evaluated the therapeutic effect of MPB-RLZ@MM NPs against AAA. Mice were infused with Ang II-pump and treated with free RLZ, bare MPB NPs, and MPB-RLZ@MM NPs for 28 days (Figure 7A). Ultrasound and anatomical analysis showed significant abdominal aortic dilation in the Ang II group (Figure 7B-C). Free RLZ, bare MPB, and MPB-RLZ@MM treatments inhibited the Ang II-induced dilation and the incidence of aneurysm formation in varying degrees (Figure 7D-E). The MPB-RLZ@MM group demonstrated the lowest arterial diameter and incidence of aneurysm formation (Figure 7D-E). The Ang II group showed characteristic features of AAA (Figure 7F) with aortic dilation (H&E), abnormal collagen deposition (Masson), and elastin degradation (EVG). The free RLZ and MPB-RLZ@MM groups had reduced F4/80^+^ macrophage infiltration and elastin degradation, possibly attributable to the anti-inflammatory effects of RLZ (Figure 7F-H). We detected the RLZ concentration in the free RLZ and MPB-RLZ@MM groups by LC-MS. The MPB-RLZ@MM group had higher RLZ concentrations at 6 and 12 h (Figure S15). In addition, the MPB-RLZ@MM group exhibited lower RLZ concentrations in non-specific organs (Figure S16), indicating reduced RLZ off-target effects. To assess VSMC loss, aortic sections were stained with a-SMA. IHC imaging (Figure S17) showed that MPB-RLZ@MM treatment effectively reduced VSMC loss. We further evaluated the therapeutic effect of MPB-RLZ@MM NPs on established small AAAs. These results showed that MPB-RLZ@MM NPs reduced aortic dilation and elastin degradation even after 2 weeks of Ang II injection (Figure S18).

### *In vivo* therapeutic mechanism

**Figure 8:**
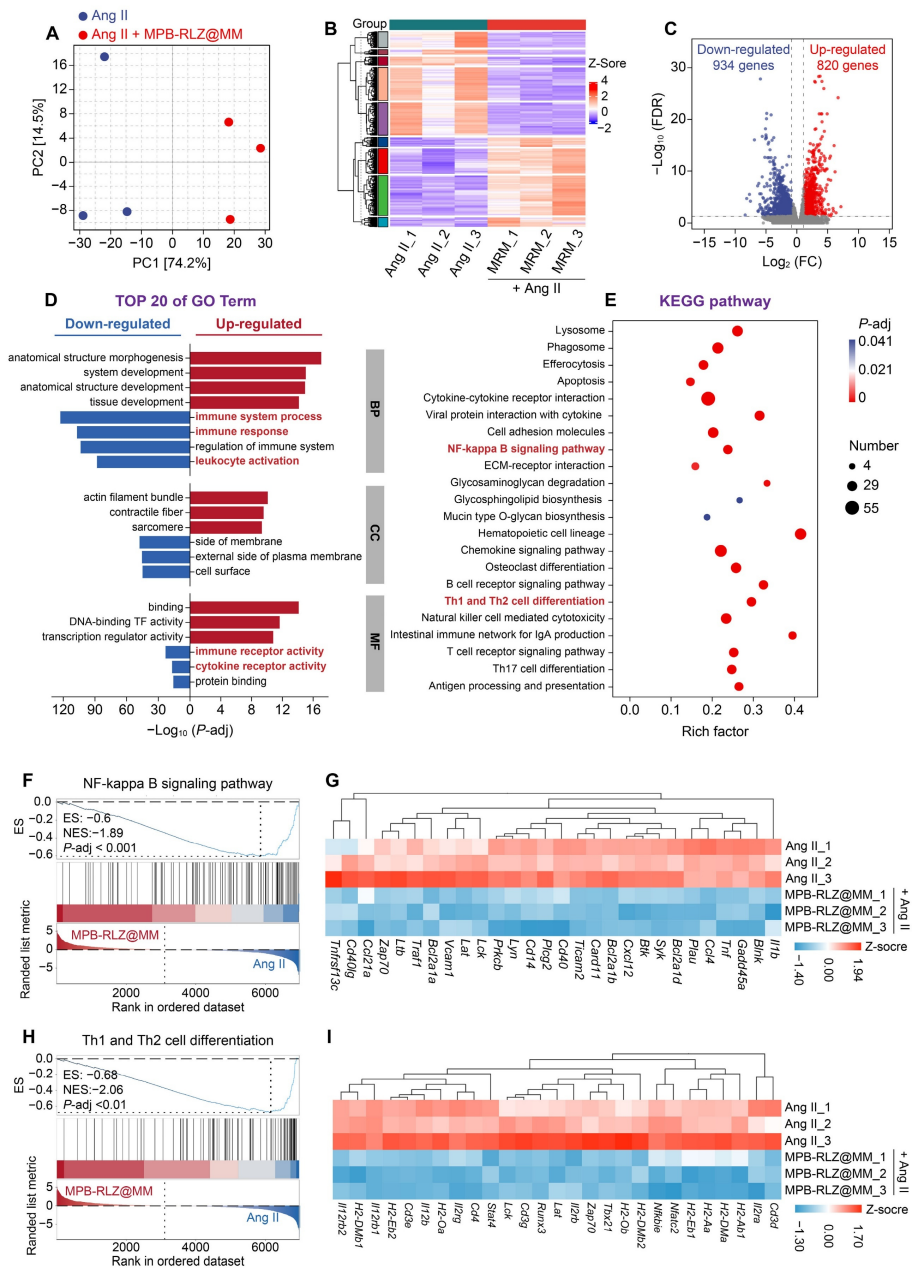
** Therapeutic mechanism by RNA-sequencing analysis.** (**A**) PCA of gene expression profiles (Ang II group vs MPB-RLZ@MM group, n = 3). (**B**) Two-way hierarchical clustering of genes and samples. (**C**) Volcano plot of up-regulated (red) and down-regulated (blue) DEGs (fold change ≥ 2 and *P*-adj < 0.05). (**D**) Top 20 enriched GO terms in BP, CC, and MF. (**E**) Significantly enriched KEGG pathways in the down-regulated DEGs. (**F**) GSEA of NF-κB signaling pathway. (**G**) Heatmap of NF-κB pathway signature genes (n = 3). (**H**) GSEA of Th1/Th2 differentiation pathway. (**I**) Heatmap of Th1/Th2 differentiation signature genes (n = 3).

PCA and clustering analysis (Figure 8A-B) showed different transcriptomic profiles between the Ang II and MPB-RLZ@MM groups. The volcano plot (Figure 8C) showed 1754 DEGs, including 820 up-regulated and 934 down-regulated genes. The prioritized lists of the top 10 up-regulated and down-regulated DEGs were listed in Table S4-5. GO enrichment analysis was used for the biological functions of DEGs. The up-regulated genes (Figure 8D) were enriched in anatomical structure morphogenesis (biological process, BP), contractile fiber (cellular component, CC), and DNA-binding transcription factor activity (molecular function, MF). The down-regulated DEGs (Figure 8D) demonstrated enrichment in immune response (BP), side of membrane (CC), and immune receptor activity (MF). KEGG pathway analysis (Figure 8E) further analyzed the potential pathways in the down-regulated DEGs. The results showed that the down-regulated DEGs were strongly associated with the NF-κB signaling pathway and Th1/Th2 cell differentiation. GSEA was used to verify these two pathways. The results demonstrated negative enrichment for both the NF-κB signaling pathway (NES = -1.89) (Figure 8F) and Th1/Th2 cell differentiation (NES = -2.06) (Figure 8H). Analysis of key gene expression confirmed that MPB-RLZ@MM treatment significantly downregulated NF-κB pathway genes (e.g., *Bcl2a1a*, *Cxcl12*, *Ccl4, Il1b* genes) (Figure 8G) and Th1/Th2 differentiation genes (e.g., *Cd3d*, *Cd4*, *Stat4*, *Tbx21* genes) (Figure 8I).

**Figure 9:**
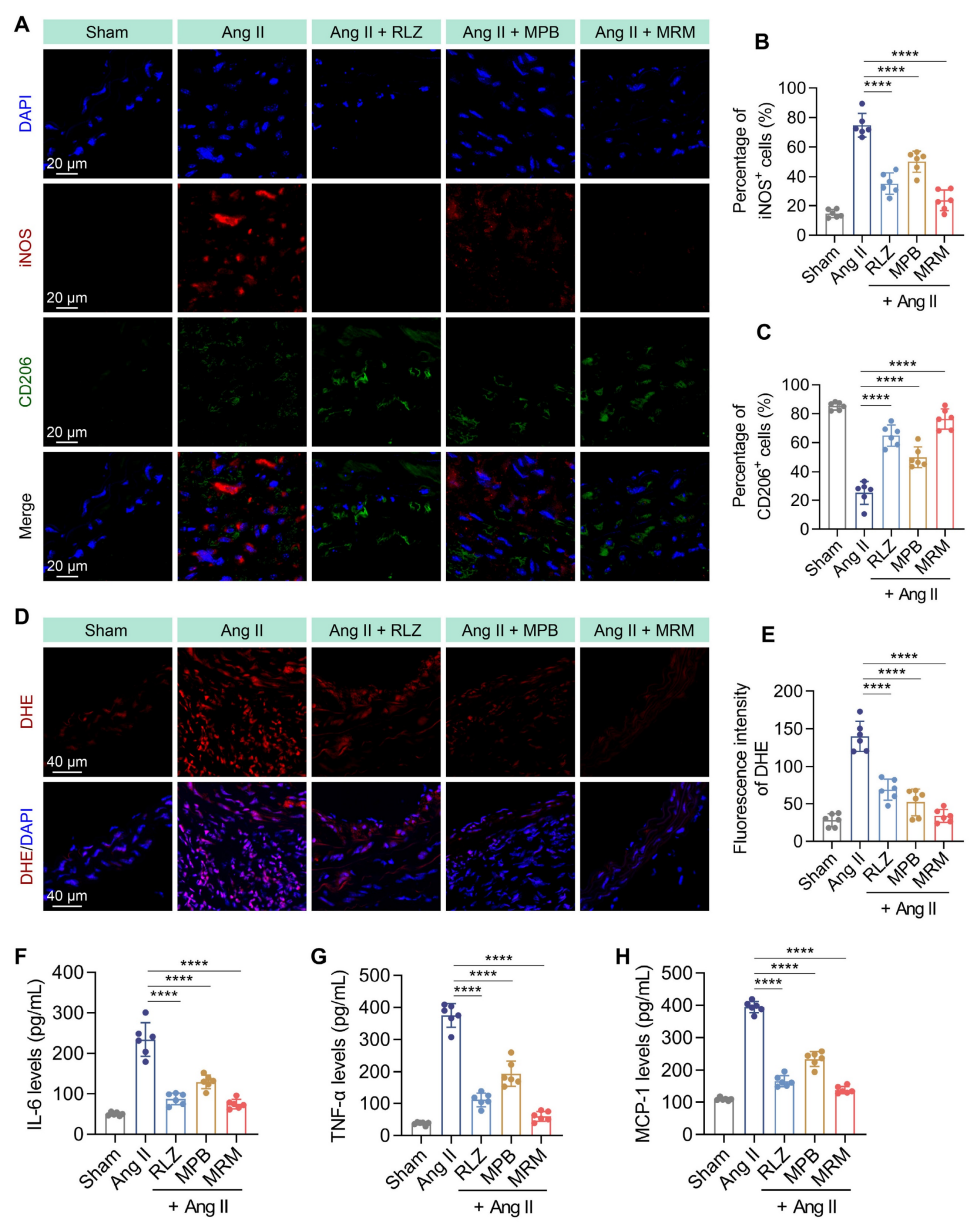
** Modulation of macrophage polarization and pro-inflammatory cytokines.** (**A**) Representative immunofluorescence images of iNOS^+^ (M1-like) and CD206^+^ (M2-like) macrophages in aortic sections. Scale bar = 20 μm. (**B-C**) Percentage of iNOS^+^ and CD206^+^ cells in aortic sections (n = 6, mean ± SD). (**D**) Representative DHE staining for ROS detection. Scale bar = 40 μm. (**E**) Quantitative analysis of ROS levels in aortic sections (n = 6, mean ± SD). (**F-H**) Pro-inflammatory cytokine levels in abdominal aortic tissues were measured by ELISA, including IL-6, TNF-α, and MCP-1 (n = 6, mean ± SD). ****p < 0.0001 as determined by ANOVA with Dunnett's multiple comparisons test.

We then evaluated the effect of MPB-RLZ@MM NPs on macrophage phenotype by immunofluorescence staining (Figure 9A). MPB-RLZ@MM treatment significantly reduced the number of M1-like macrophages (iNOS^+^ cells) while increasing the number of M2-like macrophages (CD206^+^ cells) (Figure 9B-C). DHE staining showed that MPB-RLZ@MM treatment reduced ROS levels (Figure 9D-E). ELISA confirmed that MPB-RLZ@MM treatment reduced the levels of MCP-1, IL-6, and TNF-α (Figure 9F-H).

### *In vitro* and *in vivo* biocompatibility

To assess the cytotoxicity of MPB-RLZ@MM NPs, RAW264.7 macrophages, HUVECs, and VSMCs were co-incubated with MPB-RLZ@MM NPs. Cell viabilities had no significant change, even at 200 μg/mL (Figure S19). Blood compatibility was evaluated by incubating RBCs with MPB-RLZ@MM NPs. The hemolysis phenomenon showed no red supernatant (Figure S19). Quantitative analysis further confirmed that the hemolysis rate was below 2% even at 300 μg/mL. These results show that MPB-RLZ@MM NPs exhibit excellent *in vitro* biocompatibility. Next, we evaluated the potential toxicity of MPB-RLZ@MM NPs *in vivo*. Complete blood counts (Figure S20) showed no significant changes in RBC, hemoglobin (HGB), and platelets (PLT). White blood cell (WBC) counts remained within the physiological ranges during the experiment (Figure S20). Serum biochemistry analyses indicated that key hepatic markers such as alanine aminotransferase (ALT) and aspartate aminotransferase (AST), and renal markers like blood urea nitrogen (BUN) and creatinine (CREA), remained comparable to control levels (Figure S20). H&E staining revealed no significant changes in major organs, such as inflammation or fibrosis (Figure S21). In summary, MPB-RLZ@MM NPs demonstrated excellent biosafety, with no indications of hematotoxicity and organ damage.

## Discussion

Currently, the treatments of AAA mostly rely on passive targeted strategies, such as small-molecule inhibitors and natural compounds 40-43. These strategies mainly depend on high permeability and neovascularization for drug accumulation, but their efficacy is limited. In recent years, nanomedicine has achieved tremendous progress, with several nanomedicines already approved by the FDA for clinical applications. Some preclinical studies have effectively achieved active targeted delivery to inflammation sites via surface modifications such as cRGD, P-selectin, activatable cell-penetrating peptides (ACPPs), and neutrophil membrane coating 19, 24, 43, 44. Cell membrane coating technology is an advanced biomimetic nanomedicine strategy. Leveraging the inherent properties, cell membrane-coated nanoparticles exhibit unique characteristics. As the fastest-responding immune cells in inflammation, neutrophil membranes have been widely utilized for nanoparticle delivery. In addition, macrophage membranes exhibit diverse functions and hold significant potential in the treatment of cardiovascular diseases. Liposomes and polymeric materials were used as drug delivery carriers due to their high biocompatibility. In contrast, MPB NPs demonstrated advantages such as high drug loading capacity and potent ROS scavenging ability. By leveraging the homing property of macrophage membranes, we synthesized macrophage membrane coated-MPB-RLZ NPs (MPB-RLZ@MM NPs). This nanomedicine demonstrated excellent targeting performance and therapeutic efficacy for AAA.

## Conclusions

In this study, we have successfully synthesized a novel nanomedicine designed to regulate the inflammatory response and oxidative stress for AAA. In function, MPB-RLZ@MM NPs exhibit dual effects of ROS scavenging and inflammation regulation. Overall, MPB-RLZ@MM NPs represent a promising nanomedicine strategy against AAA.

## Supplementary Material

Supplementary figures and tables.

## Figures and Tables

**Scheme 1:**
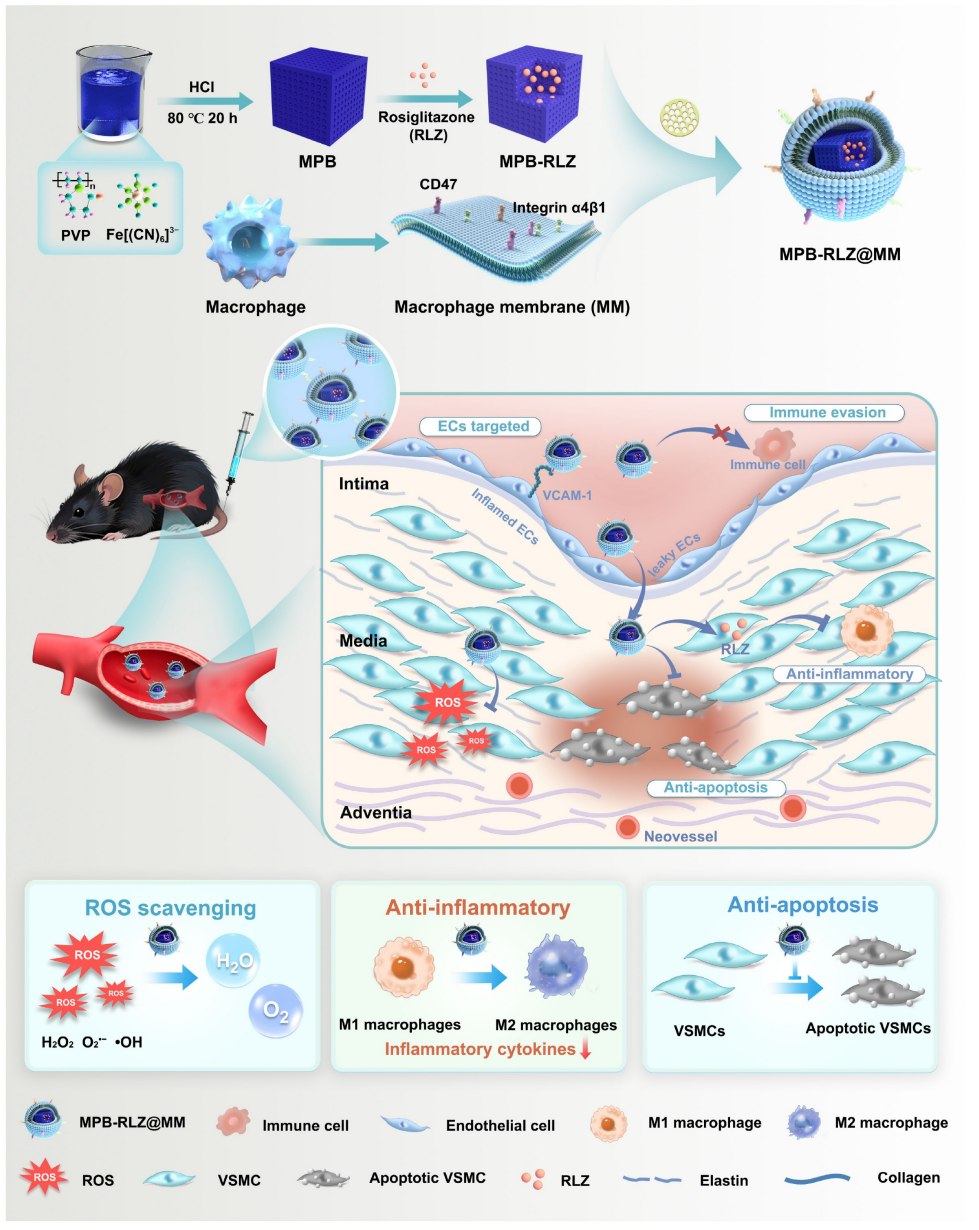
Synthesis of MPB-RLZ@MM NPs and the therapeutic efficacy against AAA. MPB-RLZ@MM NPs were synthesized by encapsulating the anti-inflammatory drug RLZ into the MPB NPs, followed by coating with macrophage membranes. The MPB-RLZ@MM NPs actively targeted AAA through the inflamed endothelium. Moreover, the MPB-RLZ@MM NPs reduced ROS levels in the vascular wall, promoted M1-like towards M2-like macrophage phenotype, and attenuated VSMC apoptosis.

## Abbreviations

AAA: abdominal aortic aneurysm
RLZ: rosiglitazone
MM: macrophage membrane
MM-NPs: macrophage membrane coated-nanoparticles
ROS: reactive oxygen species
HUVECs: human umbilical vein endothelial cells
VSMCs: vascular smooth muscle cells
MMPs: matrix metalloproteinases
MOFs: metal-organic frameworks
SOD: superoxide dismutase
CAT: catalase
GPx: glutathione peroxidase
TNF-α: tumor necrosis factor-α
IL-6: interleukin-6
IFN-γ: interferon-γ
LPS: lipopolysaccharides
SEM: scanning electron microscopy
TEM: transmission electron microscopy
PDI: polydispersity index
Rho: rhodamine
CLSM: confocal laser scanning microscopy
iNOS: inducible nitric oxide synthase
CD206: macrophage mannose receptor 1
CD86: cluster of differentiation 86
Nrf2: nuclear factor erythroid 2-related factor 2
LC-MS: liquid chromatograph mass spectrometer
DEGs: differentially expressed genes
RBC: red blood cell
HGB: hemoglobin
PLT: platelets
WBC: white blood cell
ALT: alanine aminotransferase
AST: aspartate aminotransferase
BUN: blood urea nitrogen
CREA: creatinine

## References

[1] Song P, He Y, Adeloye D, Zhu Y, Ye X, Yi Q (2023). The Global and Regional Prevalence of Abdominal Aortic Aneurysms: A Systematic Review and Modeling Analysis. Ann Surg.

[2] Sakalihasan N, Michel JB, Katsargyris A, Kuivaniemi H, Defraigne JO, Nchimi A (2018). Abdominal aortic aneurysms. Nat Rev Dis Primers.

[3] Sampson UK, Norman PE, Fowkes FG, Aboyans V, Song Y, Harrell FE Jr (2014). Estimation of global and regional incidence and prevalence of abdominal aortic aneurysms 1990 to 2010. Glob Heart.

[4] Moll FL, Powell JT, Fraedrich G, Verzini F, Haulon S, Waltham M (2011). Management of abdominal aortic aneurysms clinical practice guidelines of the European society for vascular surgery. Eur J Vasc Endovasc Surg.

[5] Wanhainen A, Van Herzeele I, Bastos Goncalves F, Bellmunt Montoya S, Berard X, Boyle JR (2024). Editor's Choice - European Society for Vascular Surgery (ESVS) 2024 Clinical Practice Guidelines on the Management of Abdominal Aorto-Iliac Artery Aneurysms. Eur J Vasc Endovasc Surg.

[6] Chaikof EL, Brewster DC, Dalman RL, Makaroun MS, Illig KA, Sicard GA (2009). SVS practice guidelines for the care of patients with an abdominal aortic aneurysm: executive summary. J Vasc Surg.

[7] Puertas-Umbert L, Almendra-Pegueros R, Jimenez-Altayo F, Sirvent M, Galan M, Martinez-Gonzalez J (2023). Novel pharmacological approaches in abdominal aortic aneurysm. Clin Sci (Lond).

[8] Gao J, Cao H, Hu G, Wu Y, Xu Y, Cui H (2023). The mechanism and therapy of aortic aneurysms. Signal Transduct Target Ther.

[9] Rodriguez PL, Harada T, Christian DA, Pantano DA, Tsai RK, Discher DE (2013). Minimal "Self" peptides that inhibit phagocytic clearance and enhance delivery of nanoparticles. Science.

[10] Parodi A, Quattrocchi N, van de Ven AL, Chiappini C, Evangelopoulos M, Martinez JO (2013). Synthetic nanoparticles functionalized with biomimetic leukocyte membranes possess cell-like functions. Nat Nanotechnol.

[11] Hu CM, Zhang L, Aryal S, Cheung C, Fang RH, Zhang L (2011). Erythrocyte membrane-camouflaged polymeric nanoparticles as a biomimetic delivery platform. Proc Natl Acad Sci U S A.

[12] Quintana RA, Taylor WR (2019). Cellular Mechanisms of Aortic Aneurysm Formation. Circ Res.

[13] Guzik B, Sagan A, Ludew D, Mrowiecki W, Chwala M, Bujak-Gizycka B (2013). Mechanisms of oxidative stress in human aortic aneurysms-association with clinical risk factors for atherosclerosis and disease severity. Int J Cardiol.

[14] Kaneko H, Anzai T, Morisawa M, Kohno T, Nagai T, Anzai A (2011). Resveratrol prevents the development of abdominal aortic aneurysm through attenuation of inflammation, oxidative stress, and neovascularization. Atherosclerosis.

[15] Dou Y, Li C, Li L, Guo J, Zhang J (2020). Bioresponsive drug delivery systems for the treatment of inflammatory diseases. J Control Release.

[16] Li C, Wu P, Dou Y, Li Q, Zhang J (2022). Bioresponsive nanoplatforms for imaging and therapy of cardiovascular diseases. View.

[17] Cheng J, Zhang R, Li C, Tao H, Dou Y, Wang Y (2018). A Targeting Nanotherapy for Abdominal Aortic Aneurysms. J Am Coll Cardiol.

[18] Mo F, Wang C, Li S, Li Z, Xiao C, Zhang Y (2024). A Dual-Targeting, Multi-Faceted Biocompatible Nanodrug Optimizes the Microenvironment to Ameliorate Abdominal Aortic Aneurysm. Adv Mater.

[19] Lin W, Hu K, Li C, Pu W, Yan X, Chen H (2022). A Multi-Bioactive Nanomicelle-Based "One Stone for Multiple Birds" Strategy for Precision Therapy of Abdominal Aortic Aneurysms. Adv Mater.

[20] Zhang W, Hu S, Yin JJ, He W, Lu W, Ma M (2016). Prussian Blue Nanoparticles as Multienzyme Mimetics and Reactive Oxygen Species Scavengers. J Am Chem Soc.

[21] Zhao Y, Song C, Wang H, Gai C, Li T, Cheng Y (2024). Polydopamine-Cloaked Nanoarchitectonics of Prussian Blue Nanoparticles Promote Functional Recovery in Neonatal and Adult Ischemic Stroke Models. Biomater Res.

[22] Zhao J, Gao W, Cai X, Xu J, Zou D, Li Z (2019). Nanozyme-mediated catalytic nanotherapy for inflammatory bowel disease. Theranostics.

[23] Cai X, Zhang K, Xie X, Zhu X, Feng J, Jin Z (2020). Self-assembly hollow manganese Prussian white nanocapsules attenuate Tau-related neuropathology and cognitive decline. Biomaterials.

[24] Shen K, Li X, Huang G, Yuan Z, Xie B, Chen T (2023). High rapamycin-loaded hollow mesoporous Prussian blue nanozyme targets lesion area of spinal cord injury to recover locomotor function. Biomaterials.

[25] Jing L, Liang X, Deng Z, Feng S, Li X, Huang M (2014). Prussian blue coated gold nanoparticles for simultaneous photoacoustic/CT bimodal imaging and photothermal ablation of cancer. Biomaterials.

[26] Wei W, Wang Y, Sun G, Chen Z, Zhang S (2025). Prussian blue nanoparticles mitigate inflammatory osteolysis by reducing oxidative stress and enhancing endogenous antioxidant systems. Colloids Surf B Biointerfaces.

[27] Ding H, Long M, Wu Y, Zhang Y, Yang Z, Yan X (2026). Subcellular distribution of Prussian blue nanozymes dictates enzymatic activity and macrophage polarization for effective colitis therapy. Biomaterials.

[28] Tian Y, Li Y, Liu J, Lin Y, Jiao J, Chen B (2022). Photothermal therapy with regulated Nrf2/NF-kappaB signaling pathway for treating bacteria-induced periodontitis. Bioact Mater.

[29] Di Y, Li H, Yang J, Feng M, Wang S, Li W (2024). PPARgamma/NF-kappaB axis contributes to cold-induced resolution of experimental colitis and preservation of intestinal barrier. Biochim Biophys Acta Mol Basis Dis.

[30] Lan T, Chen J, Zhang J, Huo F, Han X, Zhang Z (2021). Xenoextracellular matrix-rosiglitazone complex-mediated immune evasion promotes xenogenic bioengineered root regeneration by altering M1/M2 macrophage polarization. Biomaterials.

[31] Wang Y, Zhang M, Bi R, Su Y, Quan F, Lin Y (2022). ACSL4 deficiency confers protection against ferroptosis-mediated acute kidney injury. Redox Biol.

[32] Jones A, Deb R, Torsney E, Howe F, Dunkley M, Gnaneswaran Y (2009). Rosiglitazone reduces the development and rupture of experimental aortic aneurysms. Circulation.

[33] Kojima Y, Volkmer JP, McKenna K, Civelek M, Lusis AJ, Miller CL (2016). CD47-blocking antibodies restore phagocytosis and prevent atherosclerosis. Nature.

[34] Wang Y, Zhang K, Li T, Maruf A, Qin X, Luo L (2021). Macrophage membrane functionalized biomimetic nanoparticles for targeted anti-atherosclerosis applications. Theranostics.

[35] Feng H, Yin Y, Zheng R, Kang J (2021). Rosiglitazone ameliorated airway inflammation induced by cigarette smoke via inhibiting the M1 macrophage polarization by activating PPARgamma and RXRalpha. Int Immunopharmacol.

[36] Wei Z, Wang Y, Zhang K, Liao Y, Ye P, Wu J (2014). Inhibiting the Th17/IL-17A-related inflammatory responses with digoxin confers protection against experimental abdominal aortic aneurysm. Arterioscler Thromb Vasc Biol.

[37] Zhao J, Cai X, Gao W, Zhang L, Zou D, Zheng Y (2018). Prussian Blue Nanozyme with Multienzyme Activity Reduces Colitis in Mice. ACS Appl Mater Interfaces.

[38] Bai H, Kong F, Feng K, Zhang X, Dong H, Liu D (2021). Prussian Blue Nanozymes Prevent Anthracycline-Induced Liver Injury by Attenuating Oxidative Stress and Regulating Inflammation. ACS Appl Mater Interfaces.

[39] Sun J, Sukhova GK, Yang M, Wolters PJ, MacFarlane LA, Libby P (2007). Mast cells modulate the pathogenesis of elastase-induced abdominal aortic aneurysms in mice. J Clin Invest.

[40] Liu J, Liu M, Feng J, Zhu H, Wu J, Zhang H (2022). Alpha-ketoglutarate ameliorates abdominal aortic aneurysm via inhibiting PXDN/HOCL/ERK signaling pathways. J Transl Med.

[41] Sulistyowati E, Huang SE, Cheng TL, Chao YY, Li CY, Chang CW (2023). Vasculoprotective Potential of Baicalein in Angiotensin II-Infused Abdominal Aortic Aneurysms through Inhibiting Inflammation and Oxidative Stress. Int J Mol Sci.

[42] Wu Z, Zhang P, Yue J, Wang Q, Zhuang P, Jehan S (2024). Tea polyphenol nanoparticles enable targeted siRNA delivery and multi-bioactive therapy for abdominal aortic aneurysms. J Nanobiotechnology.

[43] He H, Han Q, Wang S, Long M, Zhang M, Li Y (2023). Design of a Multifunctional Nanozyme for Resolving the Proinflammatory Plaque Microenvironment and Attenuating Atherosclerosis. ACS Nano.

[44] Feng L, Dou C, Xia Y, Li B, Zhao M, Yu P (2021). Neutrophil-like Cell-Membrane-Coated Nanozyme Therapy for Ischemic Brain Damage and Long-Term Neurological Functional Recovery. ACS Nano.

