# Supplementary material for: Macrophage-biomimetic nanomedicine for targeted therapy of abdominal aortic aneurysm via Nrf2/NF-κB pathway
Source: Theranostics. 2026 Jan 1;16(7):3363–83. doi: 10.7150/thno.123428 (PMC12846727; doi:10.7150/thno.123428)
Supplement: Supplementary file 1 — Supplementary figures and tables. [file thnov16p3363s1.pdf]

Supplementary Material

Macrophage-biomimetic nanomedicine for targeted therapy of abdominal aortic aneurysm via Nrf2/NF-κB pathway

Qiujie Luo* [1, 2, 3], Wei Meng* [1, 2, 3], Jiahui Wang* [1, 2, 3], Xiang Zhang [1, 2, 3], Qingbo Meng [1, 2, 3], Mengjie Hu [1, 2, 3], Shunbo Wei [1, 2, 3], Xiaobo Yu [1, 2, 3], Dawei Deng [1, 2, 3], Yuqin Zhang [1, 2, 3], Zixuan Ma [1, 2, 3], Shentao Li [1, 2, 3], Shuang Wang [1, 2, 3], Binhao Zhang [1, 2, 3], Jingli Ding# [4], Jinping Liu# [1, 2, 3], Jianliang Zhou# [1, 2, 3]

1. Department of Cardiovascular Surgery, Zhongnan Hospital of Wuhan University,Wuhan 430071, China.
2. Hubei Provincial Engineering Research Center of Minimally Invasive Cardiovascular Surgery, Wuhan 430071, China.
3. Wuhan Clinical Research Center for Minimally Invasive Treatment of Structural Heart Disease, Wuhan 430071, China.
4. Department of Gastroenterology, Zhongnan Hospital of Wuhan University, Wuhan 430071, China.

*Equal contribution

#Corresponding author

Jianliang Zhou, MD, PhD. Email: zjl20210802@whu.edu.cn.

Jinping Liu, MD, PhD. Email: liujinping@znhospital.cn.

Jingli Ding, MD, PhD. Email: dingjingli0216@163.com.

Table S1. Primers for qPCR (Mouse).

Primer	Forward Primer	Reverse Primer
Sod1	GGGAAGCATGGCGATGAAAG	GGTTCACCGCTTGCCTTCTG
Cat	ATGGTCACCGGCACATGAAT	GCCCTGGTCGGTCTTGTAAT
Nqo1	CGCCTGAGCCCAGATATTGT	ACCACTGCAATGGGAACTGA
Il1b	TGTGCAAGTGTCTGAAGCAGC	TGGAAGCAGCCCTTCATCTT
Tnf	TGCCTATGTCTCAGCCTCTTC	GAGGCCATTTGGGAACTTCT
Nos2	GAGCGAGTTGTGGATTGTC	CCAGGAAGTAGGTGAGGG
Tgfb1	GCTGAACCAAGGAGACGGAA	ATGTCATGGATGGTGCCCAG
Il10	GCCAGAGCCACATGCTCCTA	GTCCAGCTGGTCCTTTGTTTG
Arg1	TTTTAGGGTTACGGCCGGTG	CCTCGAGGCTGTCCTTTTGA
Gapdh	ACCCTTAAGAGGGATGCTGC	CCCAATACGGCCAAATCCGT

Table S2. Elemental composition of MPB and MPB-RLZ NPs analyzed by XPS.

Samples	Element composition by XPS				
	C 1s(at%)	N 1s(at%)	O 1s(at%)	Fe 1s(at%)	S 1s(at%)
MPB	64.84	18.28	14.41	2.47	0
MPB-RLZ	64.07	18.59	13.59	2.38	1.36

Table S3. Elemental content of MPB-RLZ@MM NPs by EDS.

**Elements**	C	Fe	N	P	K	S
**Map sum spectrum (Wt%)**	86.2	9.8	3.3	0.5	0.1	0.1

Table S4. Prioritized list of the top 10 up-regulated DEGs, ranked by −$\log_{10}$ (FDR).

Gene Name	Fold Change	*P*-value	FDR (*P*-adj)	Regulation
Rgs7bp	11.0183	3.09E-33	4.84E-29	Up
Sema3c	9.5138	7.08E-33	5.54E-29	Up
Mylk	6.0937	1.5E-31	4.94E-28	Up
Myh11	13.5808	1.58E-31	4.94E-28	Up
Prkg1	6.5166	2.63E-31	6.85E-28	Up
Chmp4c	16.7605	5.17E-30	1.15E-26	Up
Hhip	81.4633	3.4E-28	6.65E-25	Up
Susd5	12.3712	5.82E-25	8.68E-22	Up
Grip2	13.4105	7.05E-25	8.68E-22	Up
Lmod1	10.0816	7.11E-25	8.68E-22	Up

Table S5. Prioritized list of the top 10 down-regulated DEGs, ranked by −$\log_{10}$ (FDR).

Gene Name	Fold Change	*P*-value	FDR (*P*-adj)	Regulation
Atp6v0d2	0.01491	3.18E-32	1.66E-28	Down
Mpeg1	0.02997	4.05E-25	7.04E-22	Down
Ctss	0.04884	1.23E-24	1.37E-21	Down
Trem2	0.02929	7.25E-24	7.56E-21	Down
Itgax	0.02615	7.45E-22	6.48E-19	Down
Gpnmb	0.02764	1.29E-20	9.62E-18	Down
Cd22	0.01829	2.66E-20	1.89E-17	Down
Slc40a1	0.0281	3.05E-20	2.07E-17	Down
Cd300a	0.06551	5.23E-20	3.27E-17	Down
Adam8	0.02887	6.42E-20	3.86E-17	Down

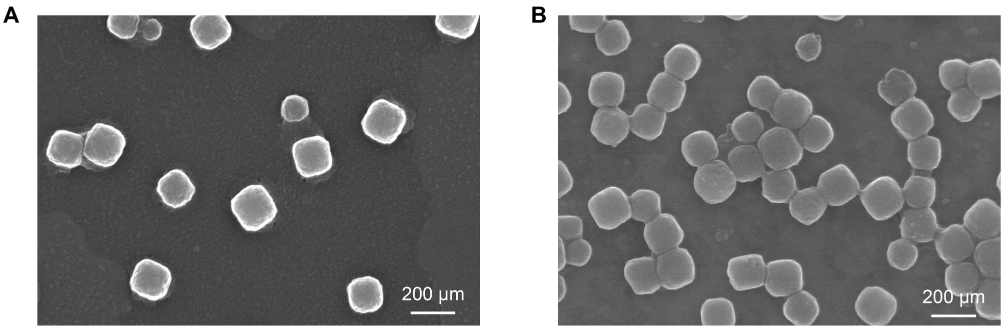

Figure S1. SEM images of MPB and MPB-RLZ NPs. (**A**) MPB NPs. (**B**) MPB-RLZ NPs. Scale bar = 200 nm.

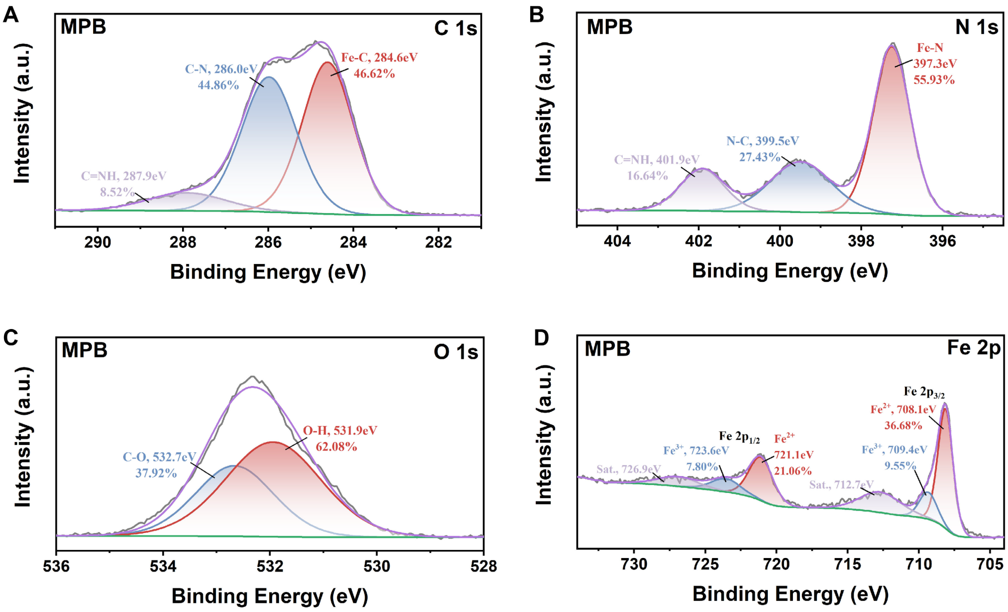

Figure S2. High-resolution XPS spectra of MPB NPs. (**A**) C 1s spectrum, (**B**) N 1s spectrum, (**C**) O 1s spectrum, (**D**) Fe 2p spectrum of MPB NPs.

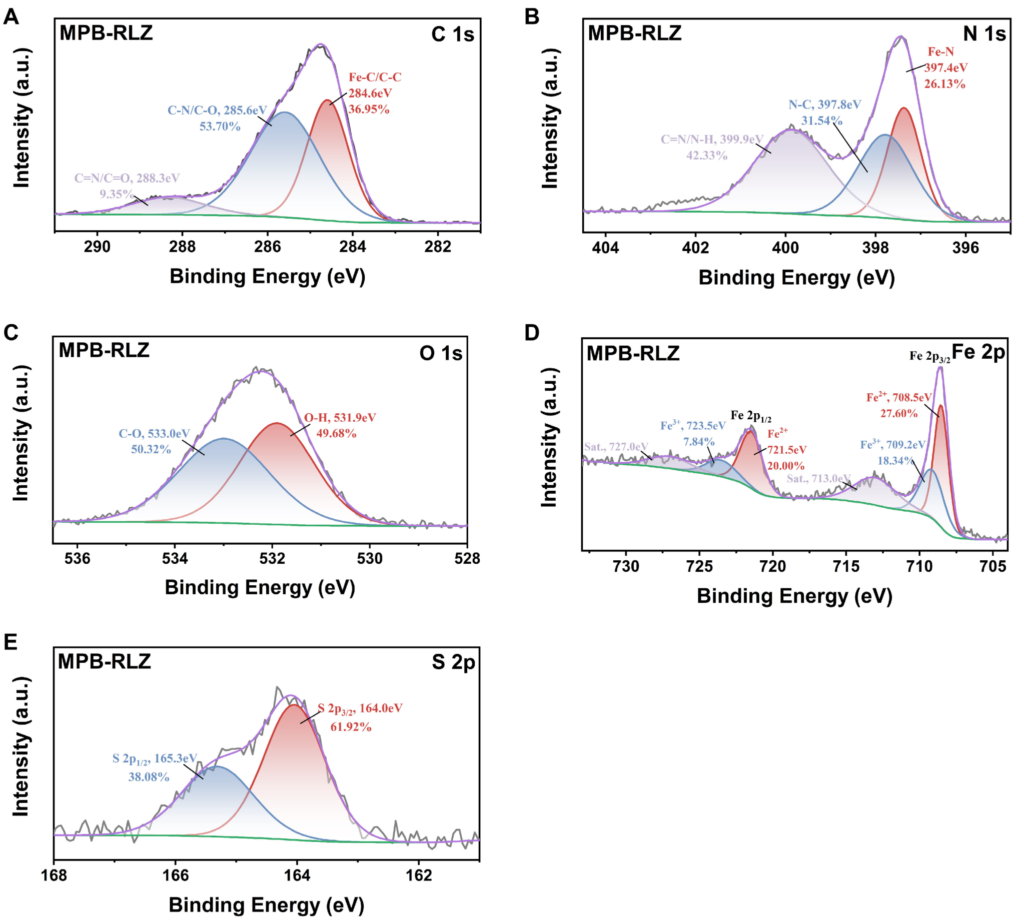

Figure S3. High-resolution XPS spectra of MPB-RLZ NPs. (**A**) C 1s spectrum, (**B**) N 1s spectrum, (**C**) O 1s spectrum, (**D**) Fe 2p spectrum, (**E**) S 2p spectrum of MPB-RLZ NPs.

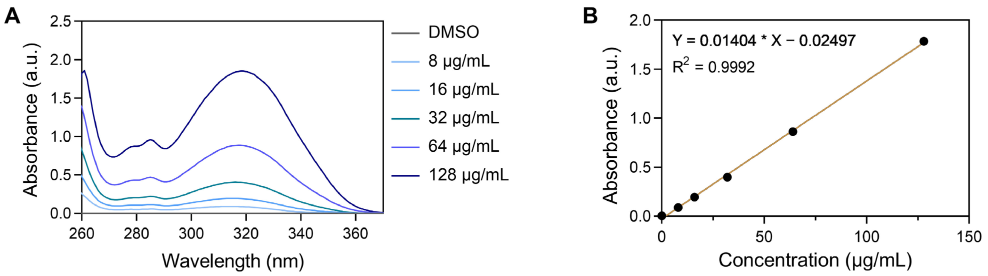

Figure S4. UV-vis spectra of RLZ and the standard curve. (**A**) UV-vis spectra of RLZ in DMSO (260-400 nm). (**B**) The standard curve of RLZ was established at 318 nm using linear regression ($Y = 0.01404 * X - 0.02497$, R^2=0.992).

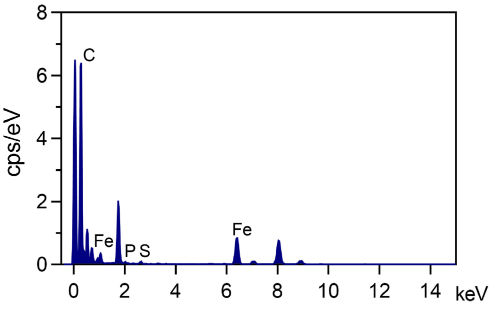

Figure S5. EDS elemental analysis of MPB-RLZ@MM NPs. Elemental content on MPB-RLZ@MM NPs.

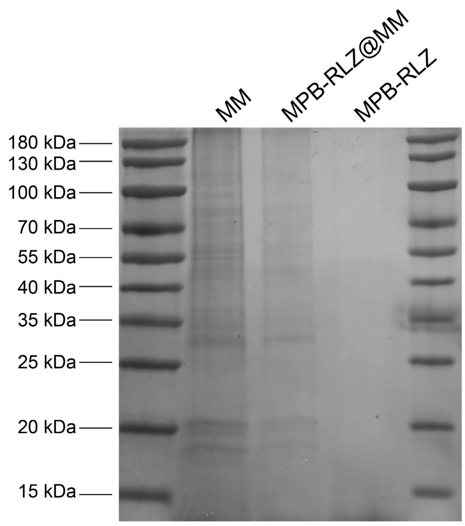

Figure S6. SDS-PAGE of MPB-RLZ@MM NPs. SDS-PAGE analysis of MM vesicles, MPB-RLZ NPs, and MPB-RLZ@MM NPs.

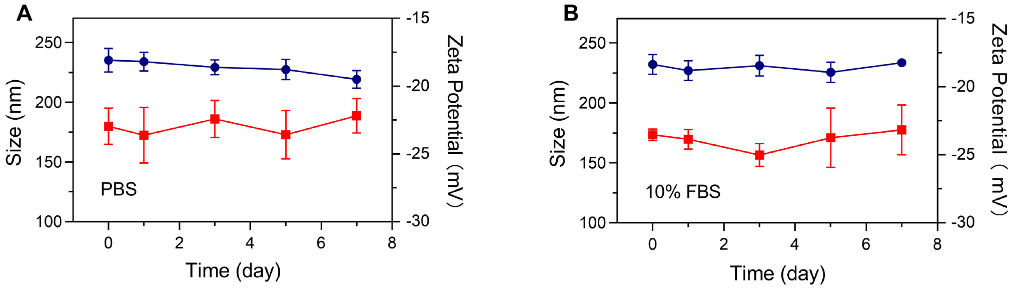

Figure S7. Size and zeta potential stability of MPB-RLZ@MM NPs. (**A-B**) Average hydrodynamic particle sizes (blue) and zeta potential (red) of MPB-RLZ@MM NPs for 1 week in PBS and 10% FBS (n = 3, mean ± SD).

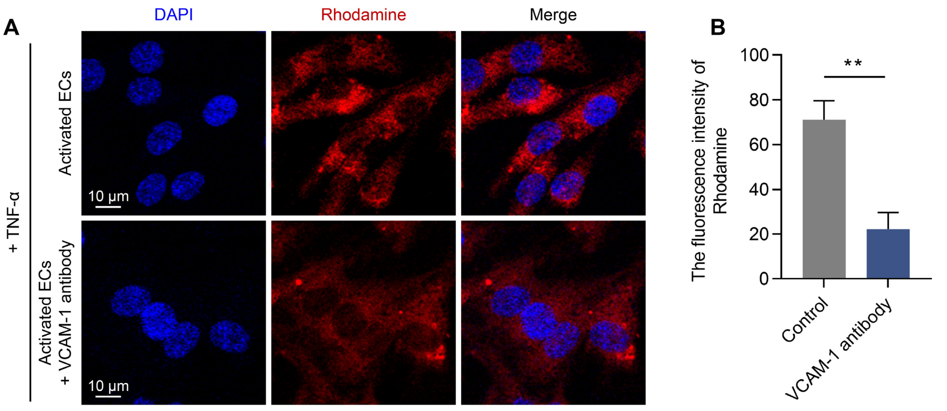

Figure S8. VCAM-1 antibody blocking assay. (**A**) Representative fluorescence (Rho) images of MPB-Rho@MM NPs binding in HUVECs with or without VCAM-1 antibody blocking. Scale bar = 10 μm. (**B**) Quantitative analysis of the Rhodamine fluorescence intensity (n = 3, mean ± SD). $P < 0.05$ was considered significant by Student's *t*-test.

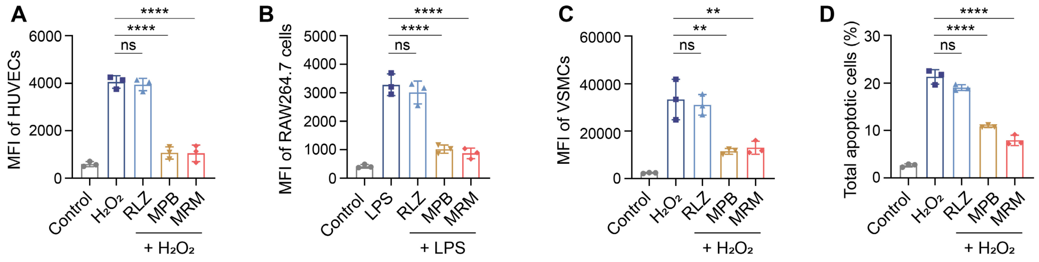

Figure S9. ***In vitro*** **ROS scavenging and VSMC apoptosis assays.** (**A-C**) Quantitative analysis from flow cytometry in HUVECs, RAW264.7 macrophages, and VSMCs (n = 3, mean ± SD). (**D**) Quantitative data in VSMC apoptosis (n = 3, mean ± SD). MRM means MPB-RLZ@MM NPs. "*ns*", no significance, $^{**}p < 0.01$ and $^{****}p < 0.0001$, as determined by ANOVA with Dunnett's multiple comparisons test.

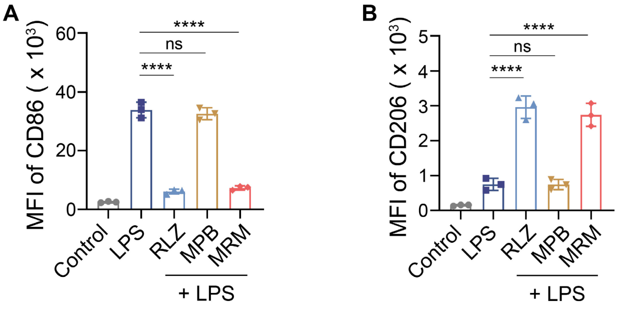

Figure S10. *In vitro* macrophage polarization assay. (**A-B**) Quantitative analysis of flow cytometry for CD86$^+$ and CD206$^+$ macrophages at varying treatment (n = 3, mean ± SD). MRM means MPB-RLZ@MM NPs. "*ns*", no significance, ****$p < 0.0001$, as determined by ANOVA with Dunnett's multiple comparisons test.

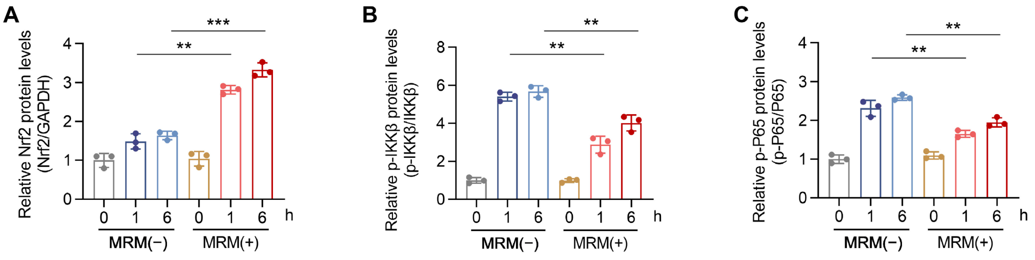

Figure S11. Protein expression levels of the Nrf2 and NF-κB pathway in LPS-induced macrophages. (**A-C**) Quantitative protein levels of Nrf2, p-IKKβ, and p-P65 (n = 3, means ± SD). MRM means MPB-RLZ@MM NPs. $P < 0.05$ was considered significant by Student's *t*-test.

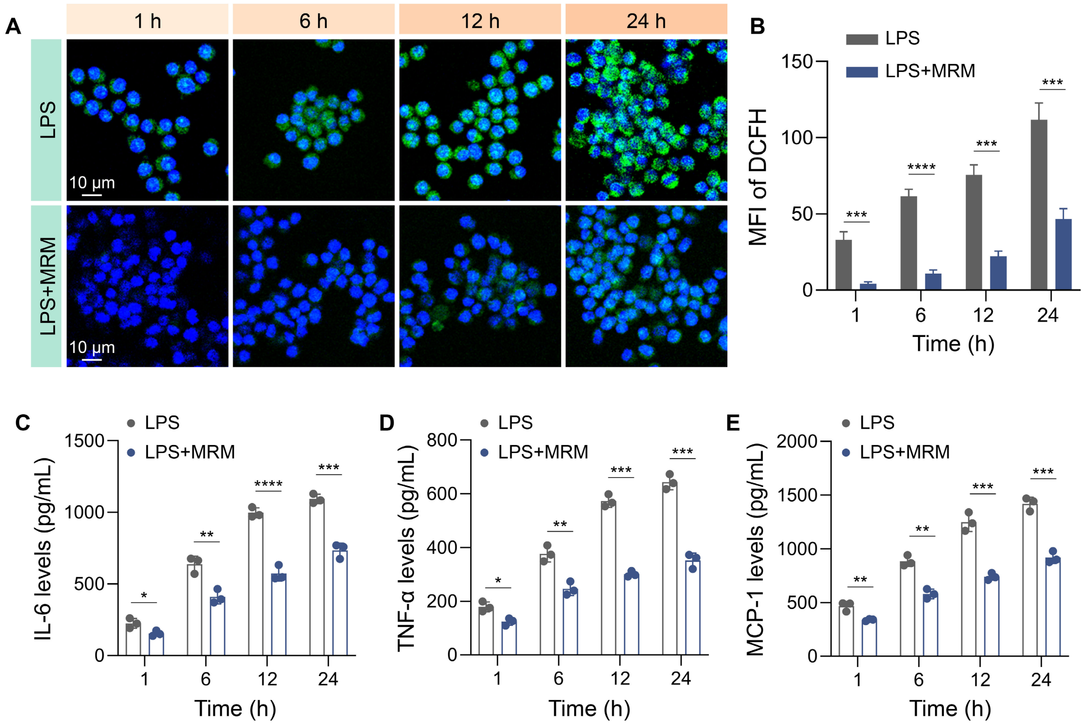

Figure S12. Temporal relationship between ROS reduction and pro-inflammatory cytokine modulation. (**A**) CLSM images of LPS/IFN-γ-induced ROS levels in RAW264.7 macrophages at different time points (1, 6, 12, and 24 h). MRM means MPB-RLZ@MM NPs. Scale bar = 10 μm. (**B**) Quantitative data from the CLSM images (n = 3, mean ± SD). (**C-E**) Levels of IL-6, TNF-α, and MCP-1 in the supernatant of RAW264.7 macrophages assessed by ELISA (n = 3, mean ± SD). $P < 0.05$ was considered statistically significant by Student's *t*-test.

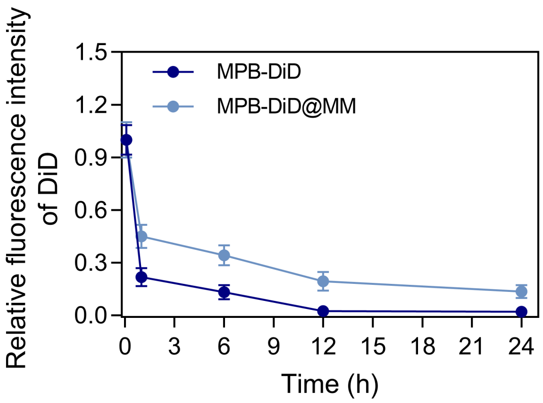

Figure S13. ***In vivo*** **pharmacokinetics of MPB-DiD@MM NPs.** Mice were

administered MPB-DiD or MPB-DiD@MM NPs. The residual DiD fluorescence intensity of plasma was quantified using a microplate reader (n = 3, mean ± SD).

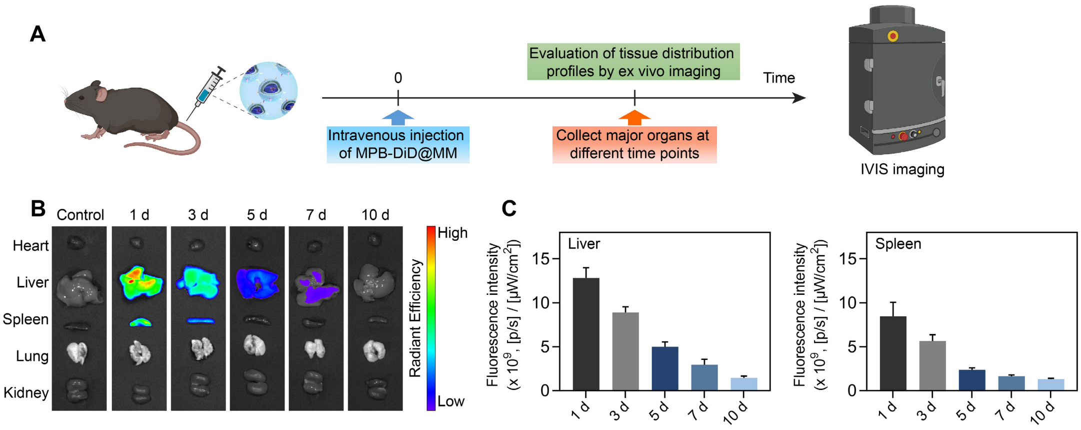

Figure S14. ***In vivo*** **tissue distribution of MPB-DiD@MM NPs.** (**A**) The schematic diagram describes the tissue distribution assay of MPB-DiD@MM NPs. (**B**) IVIS imaging showed the tissue distribution of MPB-DiD@MM NPs in major organs (heart, liver, spleen, lung, and kidney) at different time points. (**C**) Quantitative data on the fluorescence intensity of MPB-DiD@MM NPs in liver and spleen tissues (n = 3, mean ± SD).

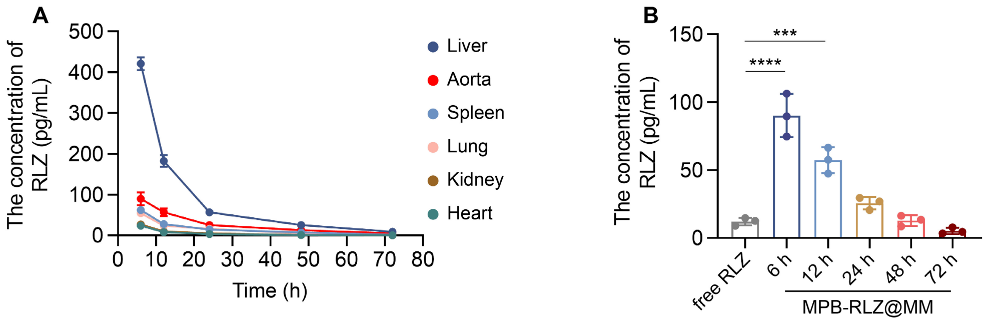

Figure S15. ***In vivo*** **RLZ concentration in MPB-RLZ@MM treatment.** (**A**) RLZ concentrations in major organs at different time points in the MPB-RLZ@MM group (n = 3, mean ± SD). (**B**) The concentrations of RLZ in the aortas in the free RLZ and

MPB-RLZ@MM groups (n = 3, mean ± SD). ***$p < 0.001$, ****$p < 0.0001$ as determined by ANOVA with Dunnett's multiple comparisons test.

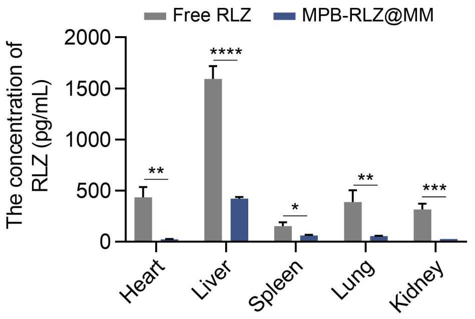

Figure S16. *In vivo* non-specific tissue distribution of RLZ. The non-specific distribution of RLZ in the free RLZ and MPB-RLZ@MM groups (n = 3, mean ± SD). $P < 0.05$ was considered significant by Student's t-test.

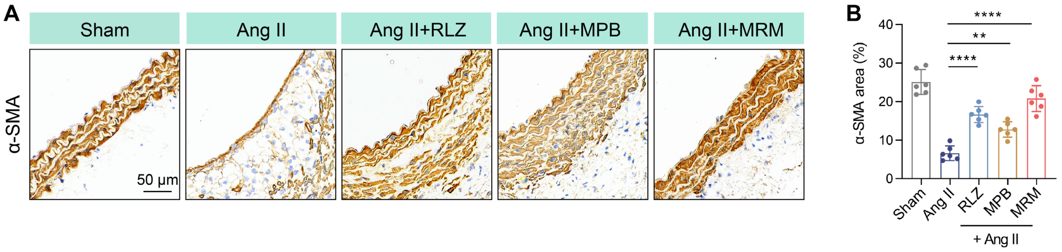

Figure S17. Immunohistochemical (α-SMA) staining. (**A**) Representative aortic α-SMA staining images following treatment with free RLZ, bare MPB NPs, and MRM (MPB-RLZ@MM) NPs. Scale bar = 50 μm. (**B**) α-SMA area (%) of the abdominal aorta (n = 3, mean ± SD). $P < 0.05$ was considered statistically significant by ANOVA with Dunnett's multiple comparisons test.

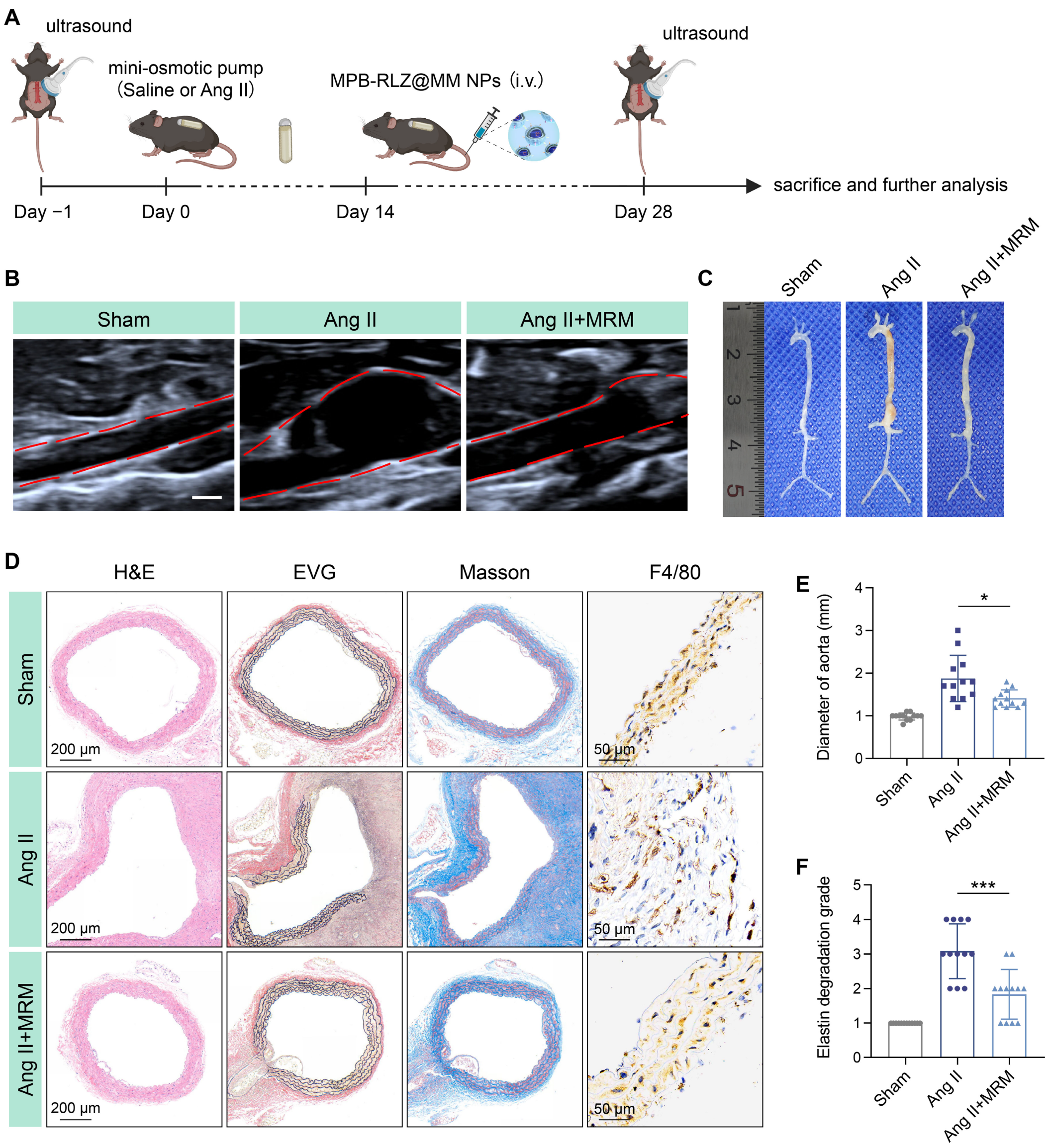

Figure S18. Therapeutic evaluation of MPB-RLZ@MM NPs in the established small AAA. (**A**) Experimental design schematic. (**B**) Representative ultrasound images of the abdominal aorta. MRM means MPB-RLZ@MM NPs. Scale bar = 1 mm. (**C**) Representative images of the aorta from the aortic arch to the iliac arteries. (**D**) Histopathological analysis of H&E, Masson, EVG, and F4/80 staining. (**E**) Maximum aortic diameter in different groups (n = 12, mean ± SD). (**F**) Elastin degradation score in aortic sections (n = 12, mean ± SD). *$p < 0.05$ and ***$p < 0.001$ as determined by Student's *t*-test.

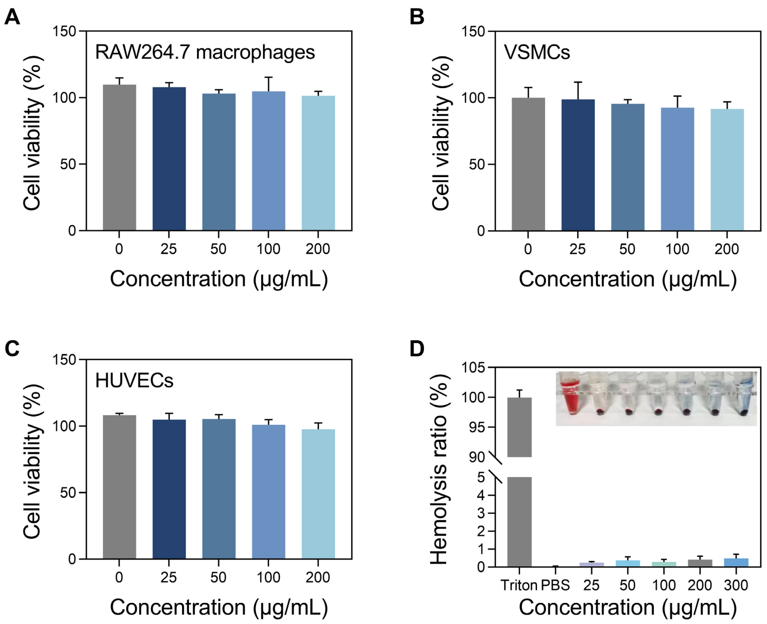

Figure S19. ***In vitro*** **cytotoxicity and hemocompatibility of MPB-RLZ@MM NPs.** (**A-C**) The cell viabilities of RAW264.7 macrophages, HUVECs, and VSMCs following incubation with various concentrations of MPB-RLZ@MM NPs for 24 h (n = 3). (**D**) Hemolysis assay of MPB-RLZ@MM NPs at various concentrations. Hemolysis photos in different groups (top panel), and the hemolysis ratio was measured at 540 nm (n = 3). Data were presented as means ± SD.

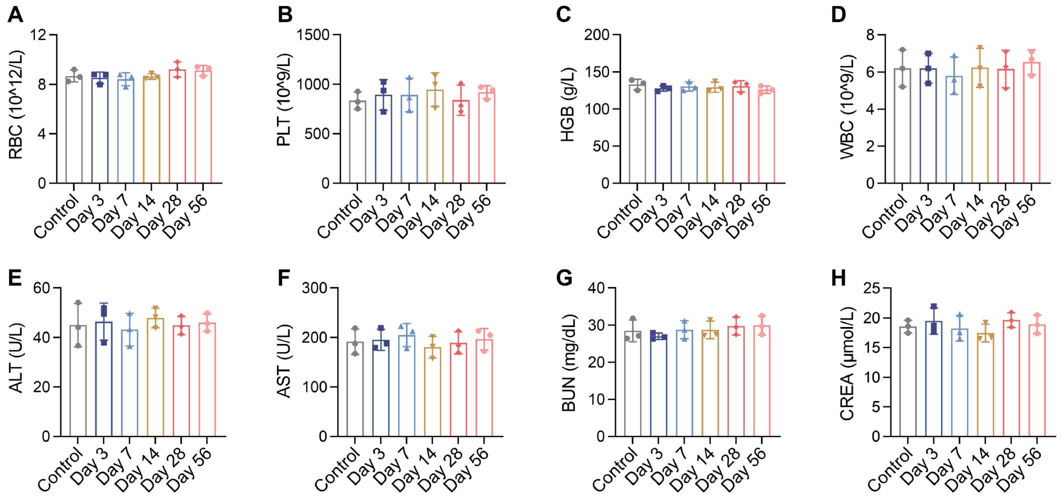

Figure S20. ***In vivo*** **blood biosafety of MPB-RLZ@MM NPs.** (**A-C**) Complete blood

counts in RBC, HGB, and PLT after treatment with MPB-RLZ@MM NPs (n = 3, mean ± SD). (**D-H**) WBC counts, hepatic markers (ALT, AST), and renal markers (BUN, CREA) in MPB-RLZ@MM treatment (n = 3, mean ± SD).

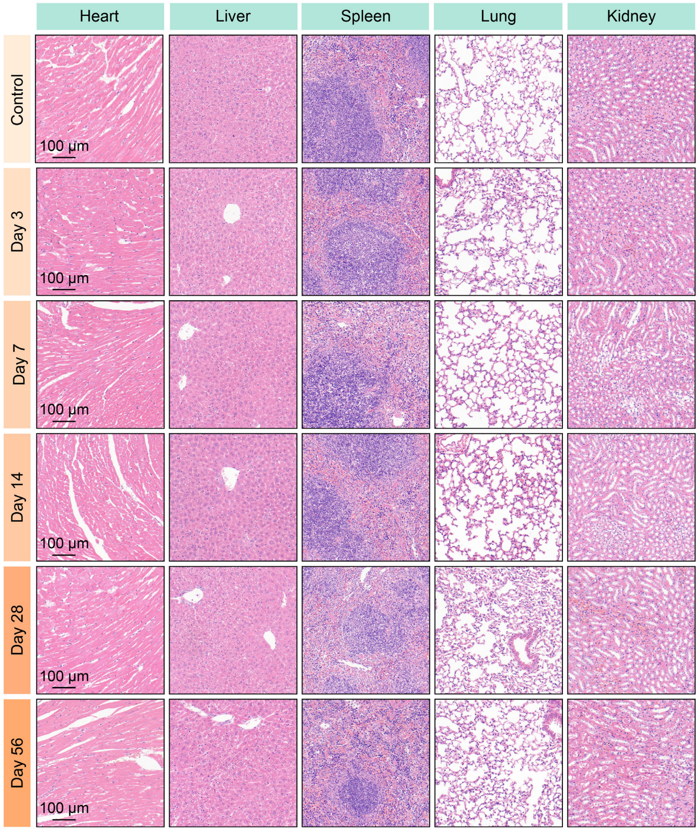

Figure S21. ***In vivo*** **organ toxicity assessment after MPB-RLZ@MM treatment.** H&E staining of major organs treated with MPB-RLZ@MM NPs at different times (n = 3). Scale bar = 100 μm.
